# Proteomics to predict relapse in patients with myelodysplastic neoplasms undergoing allogeneic hematopoietic cell transplantation

**DOI:** 10.1186/s40364-023-00550-0

**Published:** 2024-01-25

**Authors:** Guru Subramanian Guru Murthy, Tao Zhang, Yung-Tsi Bolon, Stephen Spellman, Jing Dong, Paul Auer, Wael Saber

**Affiliations:** 1 Division of Hematology & Oncology, Medical College of Wisconsin, Milwaukee, Wisconsin USA; 2 National Marrow Donor Program/Be the Match, Minneapolis, Minnesota USA; 3 Division of Biostatistics, Medical College of Wisconsin, Milwaukee, Wisconsin USA; 4 CIBMTR® (Center for International Blood and Marrow Transplant Research), Department of Medicine, Medical College of Wisconsin, Milwaukee, Wisconsin USA

**Keywords:** Myelodysplasia, Stem cell transplantation, Allogeneic, Proteomics

## Abstract

Disease relapse remains a major barrier to success after allogeneic hematopoietic cell transplantation (allo-HCT) in myelodysplastic neoplasms (MDS). While certain high risk genomic alterations are associated with increased risk of relapse, there is a lack of clinically applicable tools to analyze the downstream cellular events that are associated with relapse. We hypothesized that unique proteomic signatures in MDS patients undergoing allo-HCT could serve as a tool to understand this aspect and predict relapse. Using the Center for International Blood and Marrow Transplant Research (CIBMTR) database, we identified 52 MDS patients who underwent allo-HCT and analyzed their proteomic profile from pretransplant blood samples in a matched case-control design. Twenty-six patients without disease relapse after allo-HCT (controls) were matched with 26 patients who experienced relapse (cases). Proteomics assessment was conducted using the Slow Off-rate Modified Aptamers (SOMAmer) based assay. In gene set enrichment analysis, we noted that expression in the hallmark complement, and hallmark allograft rejection pathways were statistically enriched among patients who had disease relapse post-transplant. In addition, correlation analyses showed that methylation array probes in *cis-* and transcription regulatory elements of immune pathway genes were modulated and differentially sensitize the immune response. These findings suggest that proteomic analysis could serve as a novel tool for prediction of relapse after allo-HCT in MDS.

**Supplementary Information:**

The online version contains supplementary material available at 10.1186/s40364-023-00550-0.

## To the editor

Myelodysplastic neoplasms (MDS) are clonal hematopoietic disorders characterized by ineffective hematopoiesis and risk of transformation to acute myeloid leukemia. While allogeneic hematopoietic stem cell transplantation (allo-HCT) remains a potentially curative treatment, their long-term outcomes are suboptimal [1]. Disease relapse remains a major barrier and novel tools to predict relapse are urgently needed. Genomic abnormalities seen in MDS could translate into qualitative and quantitative alterations in downstream protein expression that may influence relapse [2, 3]. Utilization of proteomics could complement the insights gained through genomics and pave the way for an integrated multi-omics model to better understand relapse. Hence, we conducted a study to analyze the association between proteomics and relapse in MDS patients undergoing allo-HCT.

Using Center for International Blood and Marrow Transplant Research (CIBMTR) database, a retrospective matched case-control study was conducted in MDS patients who underwent allo-HCT from 2009 to 2014. Among 52 patients identified, 26 without relapse after allo-HCT (controls) were matched for 26 with relapse after allo-HCT (cases), based on age, gender, race/ethnicity, performance status, MDS therapy, IPSS-R, donor type, conditioning intensity, and transplant year (Figure S1, Supplement). Only patients with wild-type TP53, RAS pathway, and JAK2 genes were included to promote the discovery of novel risk factors. Relapse was defined as evidence of detectable MDS after allo-HCT, as reported by individual centers. Proteomic profiling was conducted with pre-transplant whole blood samples using Slow Off-rate Modified Aptamers (SOMAmer) based assay (details in Supplement) [4]. Pathway level differential expression analysis was conducted using gene set enrichment analysis (GSEA) [5]. DNA methylation signature in whole blood was investigated using Infinium MethylationEPIC array (Illumina). A predictive model with multi-omics was also constructed using iOmicsPASS approach (details in Supplement).

**Fig. 1:**
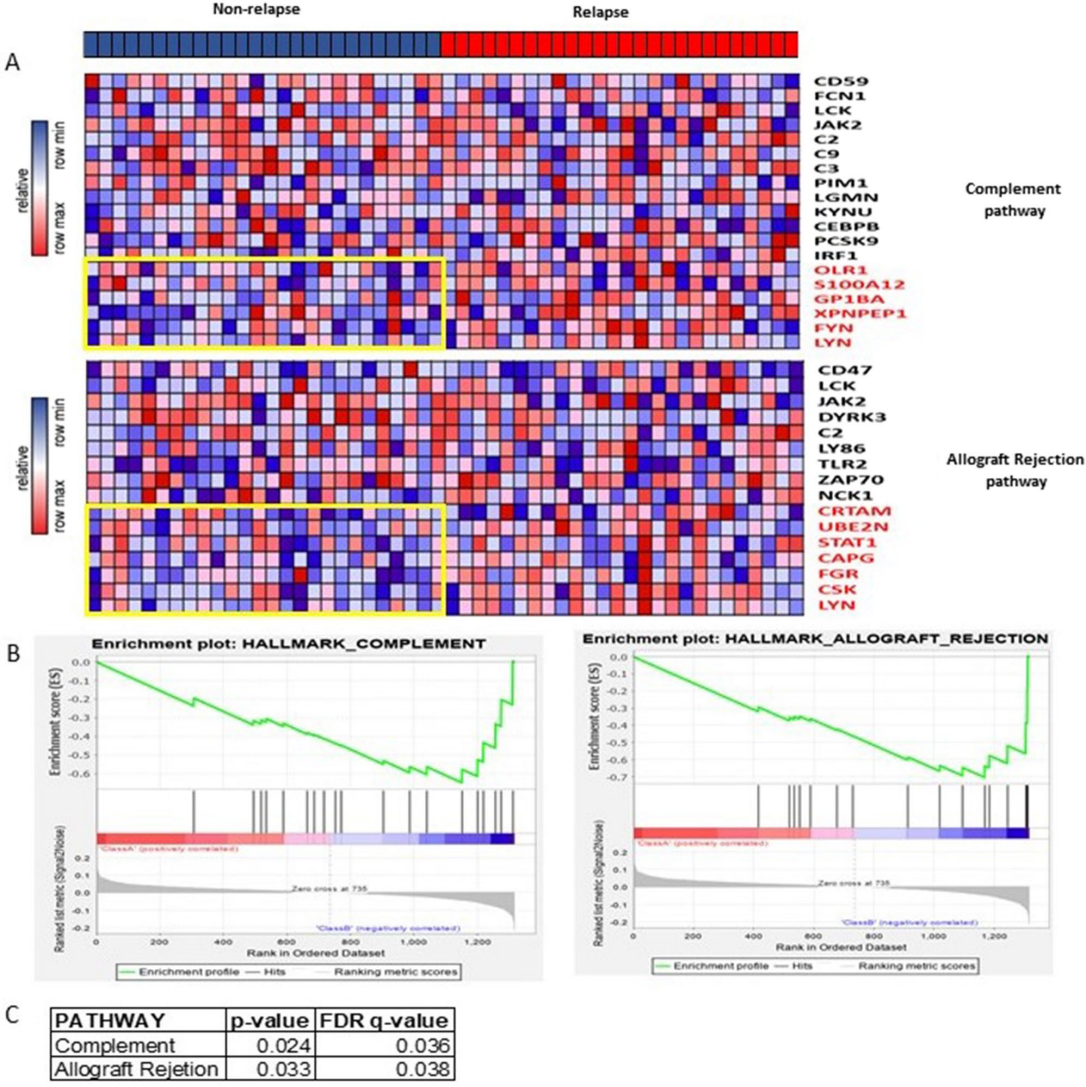
Allograft rejection and complement pathways were enriched in MDS relapse group by proteomics gene set enrichment analysis. **A** Protein expression heatmap for two representative pathways: complement pathway and allograft rejection pathway; Differentially expressed proteins between non-relapse and relapse groups are highlighted by red color. **B** The profile of ES score and positions of DE gene candidates in the rank list from GSEA leading edge analysis. **C** the normal p value and FDR q value for complement and allograft rejection pathways

Baseline characteristics are included in Table S1 (Supplementary Material 1). In proteomic analysis, under-expression or overexpression of several pathway level proteins were seen in patients with or without relapse (Fig. 1). At a single protein level, a statistically significant candidate was not identified after adjusting for variables. However, GSEA showed that hallmark complement (P = 0.024, FDR = 0.036) and hallmark allograft rejection (P = 0.033, FDR = 0.038) pathways were significantly associated with relapse after investigation of several pathways included in GSEA (Supplementary Material 2). Of note, hallmark complement pathway includes a set of genes which encodes the complement system and hallmark allograft pathway includes genes upregulated during graft rejection [6]. Immune proteins such as OLR1, S100A12, GP1BA, XPNPEP1, FLY, LYN from hallmark complement pathway, and CRTAM, UBE2N, STAT1, CAPG, FGR, CSK, LYN from hallmark allograft rejection pathway were significantly upregulated in patients with relapse.

For correlation between proteomics and methylation, we found that methylation of probes on cis-regulatory elements in a subset of immune pathway genes were correlated with protein expression that could differentially sensitize the underlying immune response. Methylation in one of two TSS (transcription starting site) probes and four of nine gene-body probes in *OLR1* gene region were highly correlated to OLR1 protein expression, while methylation in one of four TSS probes in *S100A12* gene and two of eleven gene-body probes in *CRTAM* gene were highly correlated to their own protein expression (Table S2, Supplement). The methylation levels of transcription factors were also noted to be correlated, such as methylation of the TF (transcription factor) gene *PRMD16* and multiple immune-related proteins, including FYN, LYN and GP1BA (Table S3, Supplement). In iOmicsPASS analysis integrating a multi-omics approach, multiple subnetwork edges from TP53 and AKT pathways were enriched in patients with relapse. Most enriched subnetwork edges came from the proteomic data, indicating that proteomic signals might be the main contributor for relapse prediction. However, smaller sample size limits the overall accuracy of this model (60% with 5-fold cross-validation) (Figure S2, Supplement).

While limited studies have evaluated the role of proteomics in MDS, ours is the first to examine its association with relapse after allo-HCT and no prior studies have investigated GSEA pathways in MDS to our knowledge [7, 8]. Our observation noting an association between relapse with hallmark complement and hallmark allograft rejection pathways and methylation changes in several genes including PRMD16 highlight their potential role in regulating immune dysfunction in relapse [9, 10]. Given smaller sample size, further validation in an independent large cohort would be needed as our study serves to generate hypothesis and proof of concept.

### Electronic supplementary material

Below is the link to the electronic supplementary material.

**Supplementary Material 1:** Supplement with methods and additional results including tables and figures

**Supplementary Material 2:** Gene set enrichment analysis pathways

## Data Availability

The datasets used and/or analyzed during the current study are available from the corresponding author on reasonable request.

## Declarations

### Disclaimer

The views expressed in this article do not reflect the official policy or position of the NIH, the Department of the Navy, the Department of Defense, the HRSA, or any other agency of the US Government.
