# Supplementary material for: Proteomics to predict relapse in patients with myelodysplastic neoplasms undergoing allogeneic hematopoietic cell transplantation
Source: Biomark Res. 2024 Jan 25;12:10. doi: 10.1186/s40364-023-00550-0 (PMC10809608; doi:10.1186/s40364-023-00550-0)
Supplement: Supplementary file 1 — Supplementary Material 1: Supplement with methods and additional results including tables and figures [file 40364_2023_550_MOESM1_ESM.docx]

**Supplement methods:**

Proteome assessment was conducted using the Slow Off-rate Modified Aptamers (SOMAmer) based assay [10]. While prior studies have conducted proteomic analysis using mass spectrometry, we used the SOMAmer platform to allow for simultaneous measurement of a larger scale of proteins with wider coverage that is optimized for protein biomarker discovery. The SOMAscan assay 1.3k measures over 1,300 protein analytes, providing robust and reproducible discovery of biomarker signatures [1]. The characterization of SOMAscan assay 1.3k consists of two parts, reproducibility, and reference range generation. The median total coefficients of variation (CVs) are comprised of almost equal amounts of intra-assay and inter-assay variation. The distribution of total CVs is slightly higher for plasma than for serum, with medians of 4.6% and 2.9%, respectively. Reference ranges used for the assay were determined for all analytes in normal healthy individuals. All data have been adjusted with hybridization normalization, plate scaling, median scaling, and calibration (QC CVs <0.1). The raw signal intensity values of relative fluorescence units (RFU) had been transformed in log2 scale. To adjust for the effect of matching, we used the SVA R package with pair-ID covariate adjustments on 52 recipient protein expression data. We then conducted pathway level differential expression analysis on GSEA on the batch corrected protein levels. For proteomics gene set enrichment analysis (GSEA), we followed the prior published approach from the Broad Institute and tested for gene sets from HALLMARK and GO database [2]. In the GSEA analyses, any gene sets were considered statistically associated with relapse if FDR values were less than 0.05.

To elucidate the regulatory mechanisms underlying the altered pathways in proteomic analyses, DNA methylation signatures have been assessed on their correlations with these pathway protein expressions [3]. Genome wide epigenetic data were investigated using the Infinium MethylationEPIC array (Illumina) [11]. After quality control, methylation probes were filtered based on missing rates (<3%) and variances (log(sd) > -4). Missing values were imputed to the average value for each probe. Probe intensities were inverse-normally transformed, and the probes were annotated into three categories: targeting transcription start site (TSS), gene body and UTR regions using GENCODE V19 annotation data. The correlations were examined between the expression of each pathway protein and the methylation level of the array probes. The correlation analyses primarily focus on the array probes from both cis elements on its gene region of each pathway protein and a set of transcription factors from TF2DNA database annotation [4-6]. The correlation between protein expression and methylation array probes was assessed by the Pearson correlation efficient (Coeff >= 0.4 or Coeff <= -0.4, p <0.05).

Additionally, a predictive model with multi-omics data was also constructed using the iOmicsPASS approach [16]. First, the iOmicsPASS approach handles the missing data of each -omics dataset using data filtering and the K-nearest neighbour (KNN) imputation methods. The multi-omics datasets are then integrated into a single dataset with edge-level features over the biological network such as GO, KEGG, PharmGKB, SMPDB, HumanCyc, BioCarta, EHMN, Reactome, NetPath, Pathway Interaction Database, and Wikipathways. Next, a subnetwork discovery module is carried out on the transformed data, shrinking each group's centroid towards the overall average centroid by using a soft-thresholding method. Finally, K-fold cross-validation is applied to optimize the shrinkage parameter, which minimizes the overall misclassification error rate.

Supplementary References:

1. Hensley, P., *SOMAmers and SOMAscan – A Protein Biomarker Discovery Platform for Rapid Analysis of Sample Collections From Bench Top to the Clinic.* J Biomol Tech, 2013. **24**(S5).

2. Subramanian, A., et al., *Gene set enrichment analysis: a knowledge-based approach for interpreting genome-wide expression profiles.* Proc Natl Acad Sci U S A, 2005. **102**(43): p. 15545-50.

3. Lowdon, R.F., H.S. Jang, and T. Wang, *Evolution of Epigenetic Regulation in Vertebrate Genomes.* Trends Genet, 2016. **32**(5): p. 269-283.

4. Berger, M.F., et al., *Compact, universal DNA microarrays to comprehensively determine transcription-factor binding site specificities.* Nat Biotechnol, 2006. **24**(11): p. 1429-35.

5. Jolma, A., et al., *DNA-binding specificities of human transcription factors.* Cell, 2013. **152**(1-2): p. 327-39.

6. Pujato, M., et al., *Prediction of DNA binding motifs from 3D models of transcription factors; identifying TLX3 regulated genes.* Nucleic Acids Res, 2014. **42**(22): p. 13500-12.

7. Koh, H.W.L., et al., *iOmicsPASS: network-based integration of multiomics data for predictive subnetwork discovery.* NPJ Syst Biol Appl, 2019. **5**: p. 22

**Figure S1: Schema of methodology**

**
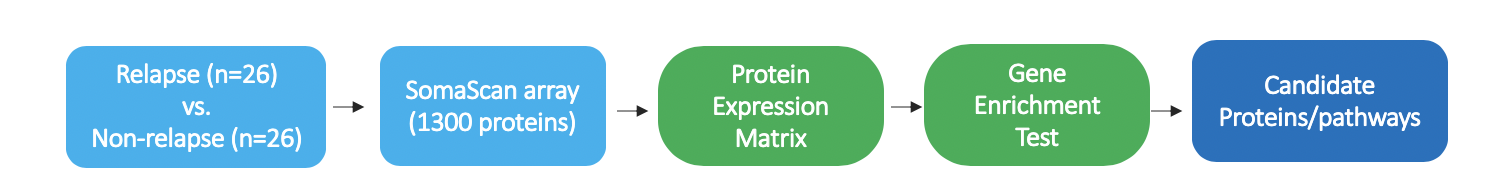
**

**Figure S2: Preliminary modeling using multi-Omics data integration** **for MDS outcome prediction.**

A. The performance comparison between iOmicsPASS and Ensemble machine learning by confusion matrix. B. Important proteomic features in TP53 / AKT signal pathways from iOmicsPASS subnetwork analysis.


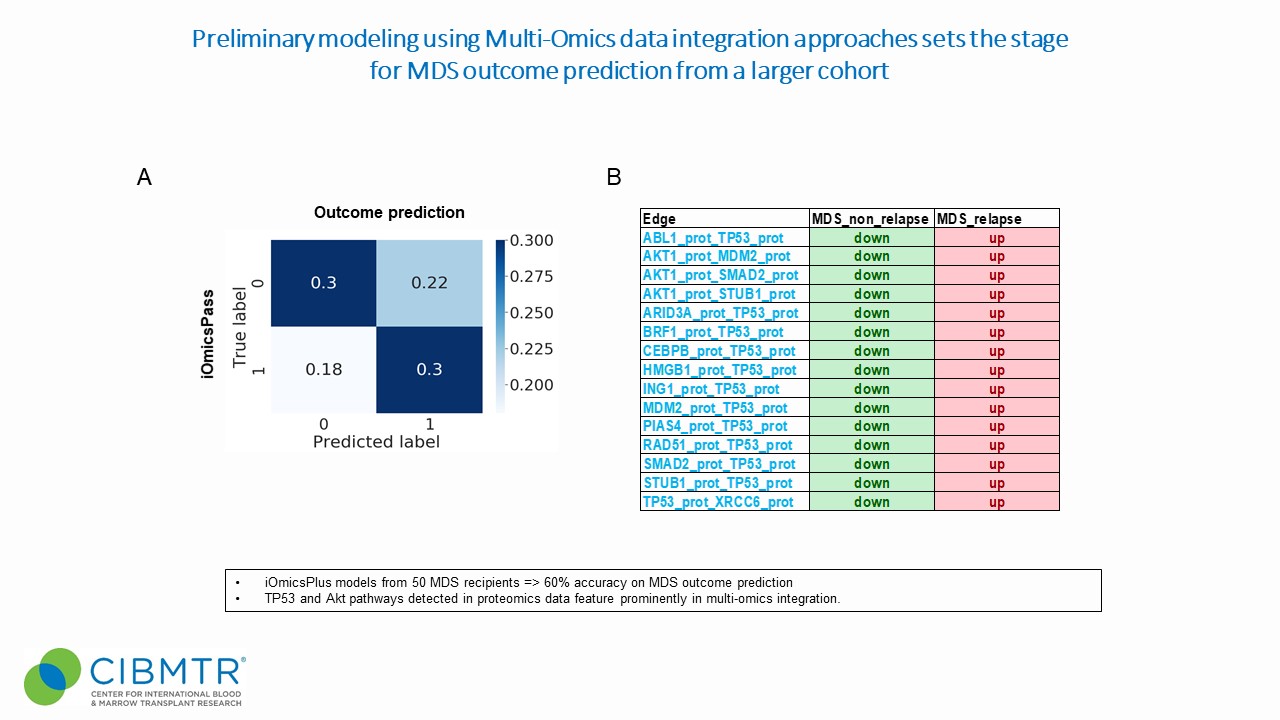


**Table S1- Baseline characteristics of study cohort**

| **Variable** | **No Relapse N(%)** | **Relapse N(%)** | **p-value** |
| --- | --- | --- | --- |
| **No. of patients** | 26 | 26 |  |
| **Patient age** |  |  | 1 |
| 50-59 years | 3(12) | 3(12) |  |
| 60 years and older | 23(88) | 23(88) |  |
| Median (min-max) in years | 63.52(53.36-69.51) | 63.59(52.95-69.79) |  |
| **Sex - no. (%)** |  |  | 0.79 |
| Male | 15(58) | 17(65) |  |
| **Race/ethnicity - no. (%)** |  |  |  |
| Caucasian, non-Hispanic | 25(96) | 25(100) |  |
| Hispanic | 1(4) | 0(0) |  |
| Missing | 0(0) | 1(4) |  |
| **Year of transplant (%)** |  |  | 0.52 |
| 2009-2010 | 2(8) | 1(4) |  |
| 2011-2012 | 14(54) | 11(42) |  |
| 2013-2014 | 10(38) | 14(54) |  |
| **Donor product source - no. (%)** |  |  | 1 |
| HLA-identical sibling | 3(12) | 3(12) |  |
| Well-matched unrelated (8/8) | 23(88) | 23(88) |  |
| **Conditioning regimen intensity- no. (%)** |  |  | 1 |
| Myeloablative | 8(31) | 8(31) |  |
| Reduced Intensity | 18(69) | 16(62) |  |
| Non-myeloablative | 0(0) | 2(8) |  |
| **Conditioning regimen - no (%)** |  |  |  |
| TBI/Flu | 5 | 3 | 0.06 |
| TBI/other(s) | 0 | 1 |  |
| Bu/Cy | 5 | 2 |  |
| Bu/Mel | 0 | 2 |  |
| Flu/Bu | 22 | 33 |  |
| Flu/Mel |  |  |  |
| **Stem cell source - no (%)** |  |  |  |
| Peripheral blood | 47 | 47 | 1 |
| Bone marrow | 0 | 0 |  |
| **IPSS-R score pre transplant - no. (%)** |  |  | 1 |
| Very low | 2(8) | 2(8) |  |
| Low | 12(46) | 12(46) |  |
| Intermediate | 8(31) | 8(31) |  |
| High | 4(15) | 4(15) |  |
| **Karnofsky Performance Score - no. (%)** |  |  | 1 |
| ≤80 | 7(27) | 7(27) |  |
| 90-100 | 18(69) | 19(73) |  |
| Missing | 1(4) | 0(0) |  |
| **Treatments prior to transplant (%)** |  |  | 0.15 |
| Hypomethylating agents alone | 12(46) | 18(69) |  |
| Chemotherapy alone | 1(4) | 0(0) |  |
| Both | 2(8) | 0(0) |  |
| Neither | 11(42) | 8(31) |  |
| Median duration of follow-up after allo-HCT (months) | 69.08(4.11-120.43) | 13.62(2.34-73.82) | NA |

**Table S2. The summary of correlation analyses between proteomics data and methylation signatures of cis elements on protein coding gene regions.**

| Proteomic candidates  from GSEA analyses | Methylation probe  in EPIC arry | Pearson coefficient | Correlation  P value | EPIC array info  (IlmnID, Name, AddressA_ID, AlleleA_ProbeSeq, AddressB_ID, AlleleB_ProbeSeq, Infinium_Design_Type, Next_Base, Color_Channel, Forward_Sequence, Genome_Build, CHR, MAPINFO, SourceSeq, Strand, UCSC_RefGene_Name, UCSC_RefGene_Accession, UCSC_RefGene_Group, UCSC_CpG_Islands_Name, Relation_to_UCSC_CpG_Island, Phantom4_Enhancers, Phantom5_Enhancers, DMR, 450k_Enhancer, HMM_Island, Regulatory_Feature_Name, Regulatory_Feature_Group, GencodeBasicV12_NAME, GencodeBasicV12_Accession, GencodeBasicV12_Group, GencodeCompV12_NAME, GencodeCompV12_Accession, GencodeCompV12_Group, DNase_Hypersensitivity_NAME, DNase_Hypersensitivity_Evidence_Count, OpenChromatin_NAME, OpenChromatin_Evidence_Count, TFBS_NAME, TFBS_Evidence_Count, Methyl27_Loci, Methyl450_Loci, Chromosome_36, Coordinate_36, SNP_ID, SNP_DISTANCE, SNP_MinorAlleleFrequency, Random_Loci) |
| --- | --- | --- | --- | --- |
| OLR1 | 94767205 | 0.5 | 0.00018 | cg21685770,cg21685770,0094767205,CTCCTCTAATACTCATAAAAAATAATTTTCCCTTTCATAAATTACTTAAC,,,II,,,TTGAGTTATGTGTATTGCAGGTGGAGAAAGTGGTGATTCTGAAGGTGTTTCAGGATATTT[CG]CTAAGTAATTTATGAAAGGGAAAACTATTTTTCATGAGCATCAGAGGAGAGATATTGTAC,37,12,10324918,CGCTAAGTAATTTATGAAAGGGAAAACTATTTTTCATGAGCATCAGAGGA,F,OLR1,NM_002543,TSS200,,,,,,,,,,C12orf59;C12orf59;OLR1;OLR1;OLR1,ENST00000381923.2;ENST00000545924.1;ENST00000432556.2;ENST00000309539.3;ENST00000545927.1,5'UTR;5'UTR;TSS200;TSS200;TSS1500,C12orf59;C12orf59;C12orf59;OLR1;OLR1;OLR1,ENST00000381923.2;ENST00000334148.7;ENST00000545924.1;ENST00000432556.2;ENST00000309539.3;ENST00000545927.1,5'UTR;5'UTR;5'UTR;TSS200;TSS200;TSS1500,chr12:10324645-10324935,3,,,,,,TRUE,12,10216185,rs12308385;rs192780902;rs61744551;rs550780427,1;37;44;48,0.009784;0.000599;0.005990;0.000200, |
|  | 2659856 | 0.46 | 0.00067 | cg21249659,cg21249659,0002659856,TCACCACTTTCTCCACCTACAATACACATAACTCAAAAATTTACRTCAAC,,,II,,,AGAAGAATGACTCAATCATTGGTAGGAGGAAGGAGAGGCTTTGCTTCATATTGGGAAGTT[CG]CTGACGCAAATTCTTGAGTTATGTGTATTGCAGGTGGAGAAAGTGGTGATTCTGAAGGTG,37,12,10324843,CGCTGACGCAAATTCTTGAGTTATGTGTATTGCAGGTGGAGAAAGTGGTG,F,OLR1,NM_002543,TSS200,,,,,,,,,,C12orf59;C12orf59;OLR1;OLR1;OLR1,ENST00000381923.2;ENST00000545924.1;ENST00000545927.1;ENST00000432556.2;ENST00000309539.3,5'UTR;5'UTR;TSS200;TSS200;TSS200,C12orf59;C12orf59;C12orf59;OLR1;OLR1;OLR1,ENST00000381923.2;ENST00000334148.7;ENST00000545924.1;ENST00000545927.1;ENST00000432556.2;ENST00000309539.3,5'UTR;5'UTR;5'UTR;TSS200;TSS200;TSS200,chr12:10324645-10324935,3,,,,,,TRUE,12,10216110,rs185281413,6,0.000599, |
|  | 76780175 | 0.42 | 0.002 | cg22212217,cg22212217,0076780175,TACTTAATTTCACAACCTTCACTTTATCTTCACCCTTCCTAAAATATACC,,,II,,,ACACTGTGTCACAAGAGTCTCCAAGAATTTTTTCTTTTCTTTTTTTTTTCCCCTTGGCCA[CG]GTACATTTTAGGAAGGGTGAAGATAAAGTGAAGGTTGTGAAATCAAGCAGGCGATAACAT,37,12,10320390,CGGTACATTTTAGGAAGGGTGAAGATAAAGTGAAGGTTGTGAAATCAAGC,F,OLR1;OLR1;OLR1,NM_002543;NM_001172632;NM_001172633,Body;Body;Body,,,,,,,,,,OLR1,ENST00000543993.1,TSS200,OLR1;OLR1;OLR1,ENST00000538873.1;ENST00000543993.1;ENST00000538745.1,TSS1500;TSS200;TSS200,chr12:10320280-10320550,3,,,,,,,12,10211657,rs137866247,30,0.000200, |
|  | 62768845 | 0.48 | 0.00034 | cg05008701,cg05008701,0062768845,TAAACCTTTAATCATCTTATAACAAAATCAAAACTACCTTCAAACCAAAC,,,II,,,AACATGTTAAGGGTCAAATGTCTCAGTTCCTGAATTTTGATGGTGCAATACAAAGCAGAC[CG]CTTGGTTTGAAGGCAGCTTTGATCCTGTTATAAGATGATCAAAGGCTCAAAAGATTTTTC,37,12,10320507,GAGCCTTTGATCATCTTATAACAGGATCAAAGCTGCCTTCAAACCAAGCG,F,OLR1;OLR1;OLR1,NM_002543;NM_001172632;NM_001172633,Body;Body;Body,,,,,,,,,,OLR1,ENST00000543993.1,TSS1500,OLR1;OLR1;OLR1,ENST00000538873.1;ENST00000543993.1;ENST00000538745.1,TSS1500;TSS1500;TSS1500,chr12:10320280-10320550,3,,,,,,,12,10211774,,,, |
|  | 10778837 | 0.52 | 9e−05 | cg19518069,cg19518069,0010778837,CCTAAACAACTCAATTCTACAACCTAAATTCACAAACAATTCAAATAAAC,,,II,,,ATCCTGGGTAGCCTAAACAACTCAGTTCTACAACCTAAATTCACAGGCAGTTCAGGTGAA[CG]TTGGGCACCATTTTCTTTCCTTCTTGGCCACTCTGGGCCATCTTTTCACTTCTTTTTTTC,37,12,10320770,CTAAACAACTCAGTTCTACAACCTAAATTCACAGGCAGTTCAGGTGAACG,R,OLR1;OLR1;OLR1,NM_002543;NM_001172632;NM_001172633,Body;Body;Body,,,,,,,,,,OLR1,ENST00000543993.1,TSS1500,OLR1;OLR1;OLR1,ENST00000538873.1;ENST00000543993.1;ENST00000538745.1,TSS1500;TSS1500;TSS1500,,,,,chr12:10320075-10320844,6,,,12,10212037,rs544625681,1,0.000200, |
| S100A12 | 16704837 | 0.45 | 0.00079 | cg02901136,cg02901136,0016704837,AAACCTAAACCCTAACTCAAAACTAAAAAAAATTCAAAACACCCCTATAC,,,II,,,CCAGACTGAATTTTGCCCTCCTGCTCAGCCTGAAACAAAATCACCCCCACTCCACCCACC[CG]CACAGGGGTGCCCTGAACCTTTTTTAGCCCTGAGCCAGGGCCCAGGTTTCAGGTTTAGCT,37,1,153348305,AACCTGGGCCCTGGCTCAGGGCTAAAAAAGGTTCAGGGCACCCCTGTGCG,F,S100A12,NM_005621,TSS1500,,,,,,,,,,S100A12;S100A12,ENST00000368737.3;ENST00000368736.1,TSS200;TSS1500,S100A12;S100A12,ENST00000368737.3;ENST00000368736.1,TSS200;TSS1500,,,chr1:153347902-153348424,4,chr1:153347878-153348494,6,,TRUE,1,151614929,rs569206892;rs552934106,5;21,0.000200;0.005990, |
| CRTAM | 36733906 | 0.36 | 0.0086 | cg07115032,cg07115032,0036733906,ACAATAACAACCATCTACCAAAATACACATAAACAAAACCAATACAATTC,,,II,,,GTAGAGGTAAAGATTGCACATGAGAACTTCCAAAATCTTGGGAAAGACCAAAAAATCTTA[CG]AACTGCACTGGTTTTGTCTATGTGTACTTTGGTAGATGGCTGTTACTGCTGCTTGTCCTT,37,11,122713816,CGAACTGCACTGGTTTTGTCTATGTGTACTTTGGTAGATGGCTGTTACTG,F,CRTAM,NM_019604,Body,,,,,,,,,,,,,,,,,,chr11:122713745-122715043,6,,,,,11,122219026,rs531167431,1,0.000399, |
|  | 13604846 | 0.4 | 0.0029 | cg14282656,cg14282656,0013604846,ATAATTTAATCCTCATATTACTACCCTAACTCCRAAACAAATCATTATCC,,,II,,,AGGAGCTTAATTGCTGAGACATTAATAATGACCTCTTAGTGCAATGCAAGATGGTGTCCT[CG]GATAATGATCTGCCCCGGAGCTAGGGCAGCAACATGAGGACCAAACCATGCACATAAAGC,37,11,122742237,CGGATAATGATCTGCCCCGGAGCTAGGGCAGCAACATGAGGACCAAACCA,F,CRTAM,NM_019604,3'UTR,,,,,,,,,,CRTAM;CRTAM,ENST00000533709.1;ENST00000227348.4,3'UTR;3'UTR,CRTAM;CRTAM,ENST00000533709.1;ENST00000227348.4,3'UTR;3'UTR,,,,,,,,TRUE,11,122247447,rs116506147,31,0.000799, |
|  | 2630929 | 0.35 | 0.01 | cg22512531,cg22512531,0002630929,ACTAATATACCCTTAAAAAACAAAACCTAATAAACTTATTTCATCTTACC,,,II,,,CAATGGCTAAGGAGTATAGAAAGGATCATTATAGTGTGTGTCTCTGTGGGTCCTATGTTA[CG]GCAAGATGAAACAAGCTTATTAGGCTCTGTCTTTTAAGGGCATACCAGTTGAAAGAGCAT,37,11,122709551,CGGCAAGATGAAACAAGCTTATTAGGCTCTGTCTTTTAAGGGCATACCAG,F,CRTAM,NM_019604,Body,,,,,,,,,,,,,,,,,,,,,,TRUE,TRUE,11,122214761,rs144316593;rs59819931,0;51,0.005391;0.016174, |
| FYN | 82707370 | − 0.48 | 0.00028 | cg24741666,cg24741666,0082707370,CTATAAAAATCAAACRAACACRTCCAACCATAACCTAAACACAAACAACC,,,II,,,GCGCACGGTGTCTGTGGAGGTCAGGCGGACACGTCCAGCCATGGCCTGGACACAGACAGC[CG]GGCTGTGTGGCACAGGACTGCCTGCTGGAAGCTTCCGCCTGCGCACTCCTCCCGGCCTCC,37,1,3038707,TGTGGAGGTCAGGCGGACACGTCCAGCCATGGCCTGGACACAGACAGCCG,R,PRDM16;PRDM16,NM_022114;NM_199454,Body;Body,chr1:3038067-3038343,S_Shore,,,,,1:3027842-3028807,,,,,,,,,,,chr1:3030640-3044810,6,,,,TRUE,1,3028567,,,, |
|  | 98652496 | − 0.45 | 0.00073 | cg10442735,cg10442735,0098652496,AATACCCACRTATACTATATATAAAAATATATCTAAATATTTTCCTACTC,,,II,,,CTCCTGGGCCCCGTTAGGGACGCAATGAGGACCCGTTTTCTGCGGGACAGAGGGTTTGGC[CG]AGCAGGAAAACATTTAGACACACCCCCACACACAGCACACGTGGGCATCAGGGCTCTGCT,37,1,3062633,ATGCCCACGTGTGCTGTGTGTGGGGGTGTGTCTAAATGTTTTCCTGCTCG,F,PRDM16;PRDM16,NM_022114;NM_199454,Body;Body,chr1:3059050-3059268,S_Shelf,,,,,1:3052292-3052708,,,,,,,,,,,chr1:3060367-3066903,6,,,,TRUE,1,3052493,rs377194829;rs180885402;rs530307548,24;41;42,0.000200;0.000799;0.000599, |
|  | 10660204 | − 0.47 | 0.00047 | cg09335331,cg09335331,0010660204,AATATTCTTAATCATTCTCTCTCCTTTTTTCTAAAATTCCTTATATACCC,,,II,,,ACGTTTTTAGCCCAAGAATGCCATGAAATCCTGCACTATCTTCGGCCCGGCTCGGAGGCA[CG]GGTATATAAGGAATTTTAGAAAAAAGGAGAGAGAATGACTAAGAATACCTCCCTGATGGC,37,1,3111908,CGGGTATATAAGGAATTTTAGAAAAAAGGAGAGAGAATGACTAAGAATAC,F,PRDM16;PRDM16,NM_199454;NM_022114,Body;Body,chr1:3111579-3111909,Island,,chr1:3111769-3111769,,,1:3101029-3101769,,,,,,,,,chr1:3111765-3111995,3,,,,,,,1,3101768,,,, |
|  | 60743243 | − 0.47 | 5e−04 | cg05641535,cg05641535,0060743243,TCRAATTCTTACCCAAATAAAAAACCCTAAAAACTACTCAATTAATCCTC,,,II,,,ACTAAACGCTCCTGGGAGGCCCTAGTGAAGCATCACAGACCATTAGACCGGGGCTTCCGC[CG]AGGACTAATTGAGCAGCCCTTAGGGCTTTTCATCTGGGCAAGAATCCGAACTCCTCCAGG,37,1,3026154,CGGATTCTTGCCCAGATGAAAAGCCCTAAGGGCTGCTCAATTAGTCCTCG,F,PRDM16;PRDM16,NM_022114;NM_199454,Body;Body,chr1:3028179-3028557,N_Shelf,,,,,,1:3026046-3026158,Unclassified_Cell_type_specific,,,,,,,chr1:3025905-3026415,3,,,,,,TRUE,1,3016014,rs572490825;rs545337043,26;45,0.000200;0.001198, |
|  | 90782987 | − 0.47 | 0.00048 | cg03844268,cg03844268,0090782987,CTATTTACCACAATAACTATACCATTTCACATCCCRAAATACCTATATTC,,,II,,,GAACCTCCAGACTGTTTACCACAGTGGCTATACCATTTCACATCCCGGGGTGCCTGTGTT[CG]TTTGACTCAGAGCTGCTCAGAAACGAGGAGCGGCCTCACAGAGCGGCTGAAGGGGCGGCC,37,1,3184187,CGAACACAGGCACCCCGGGATGTGAAATGGTATAGCCACTGTGGTAAACA,R,PRDM16;PRDM16,NM_199454;NM_022114,Body;Body,chr1:3182821-3183216,S_Shore,,,,,1:3173758-3174684,,,PRDM16,ENST00000512462.1,5'UTR,PRDM16;PRDM16,ENST00000512462.1;ENST00000463591.1,5'UTR;5'UTR,chr1:3184045-3184255,3,,,,,,,1,3174047,rs182289293;rs564678747;rs137978473,40;35;30,0.001797;0.000200;0.001597, |
|  | 90774563 | − 0.49 | 0.00027 | cg14030836,cg14030836,0090774563,TAAATTCCCTCACAACAAACTCAAAAAACCRCTTTACCAAACAAAACCTC,,,II,,,ACGCGTGGGGTGCCCACTTCAAATGTGCAGAACCAGGAGCAGCCTCTGCTCTGCCCACCT[CG]AGGCCCTGCTTGGCAAAGCGGCTTTCTGAGCCTGCTGTGAGGGAACCCACCAGCCCCGCG,37,1,2992620,CGAGGCCCTGCTTGGCAAAGCGGCTTTCTGAGCCTGCTGTGAGGGAACCC,F,PRDM16;PRDM16,NM_022114;NM_199454,Body;Body,chr1:2990030-2990718,S_Shore,,,,,1:2982111-2982672,1:2992545-2992737,Unclassified_Cell_type_specific,,,,,,,chr1:2992600-2992975,3,,,,,,TRUE,1,2982480,rs180711049,51,0.000200, |
|  | 81665904 | − 0.48 | 0.00028 | cg03157741,cg03157741,0081665904,CAAAAAAATATCCTAACTCAAAATAACAAACAAAAACCCACCCRTAAACC,,,II,,,AATGCAGTCACCAGGGAGGTGTCCTGGCTCAGGATAGCAGACAGGGACCCACCCGTAAAC[CG]TGTTGTGCTGCTTTAAATTCAGCAGGATTTTTTCTTCTTTTCCCATGAGTTTTTATTCAC,37,1,3285226,CGGTTTACGGGTGGGTCCCTGTCTGCTATCCTGAGCCAGGACACCTCCCT,R,PRDM16;PRDM16,NM_199454;NM_022114,Body;Body,,,,,,,,,,PRDM16,ENST00000512462.1,5'UTR,PRDM16;PRDM16,ENST00000512462.1;ENST00000463591.1,5'UTR;5'UTR,chr1:3285165-3285350,3,,,,,,,1,3275086,rs541699597,8,0.000200, |
|  | 50741930 | − 0.46 | 0.00069 | cg11727198,cg11727198,0050741930,AACAATAACATACATACRTATCTAAACACATATACACACAAACACAAATC,,,II,,,ACATGTGTGCAAACAGTGACATGCATGCGTGTCTAGGCACATGTACACACAAACACAGGT[CG]TGCATGCAAAACACATGTGCACACAGATGTCCACACACATATACAATTTCCTCATGGAGA,37,1,3138369,CGACCTGTGTTTGTGTGTACATGTGCCTAGACACGCATGCATGTCACTGT,R,PRDM16;PRDM16,NM_199454;NM_022114,Body;Body,,,,,,,,,,,,,,,,,,,,chr1:3138216-3139274,6,,,1,3128229,rs557124305;rs116020406,35;1,0.000200;0.004593, |
|  | 47763189 | − 0.46 | 0.00069 | cg12149490,cg12149490,0047763189,ACAATTCATAAAAATAATCTACTTAAAACCATTATACTACCTTACCATCC,,,II,,,TCCAACCAGAAGCAATTCATAAAAATGATCTGCTTAAAACCATTGTGCTGCCTTGCCATC[CG]TTGTCAAAGGTGTGAGGGGTGTGTGTGCACATGTGTTCAGTGTGGGGTGTGCGCACATGT,37,1,3062980,CGGATGGCAAGGCAGCACAATGGTTTTAAGCAGATCATTTTTATGAATTG,R,PRDM16;PRDM16,NM_199454;NM_022114,Body;Body,chr1:3059050-3059268,S_Shelf,,,,,,,,,,,,,,,,chr1:3060367-3066903,6,chr1:3062918-3063852,4,,,1,3052840,rs567083445,1,0.000200, |
|  | 59788837 | − 0.48 | 0.00035 | cg05573412,cg05573412,0059788837,ATACATACACATATATATAACCTCACATACATATACAACCCCCCAAAAAC,,,II,,,GTGTGTGTGTATGTACGTTTGTGTGTGAGTGCAAATGTGTGTGAGAGTGTTTGGAGGTCA[CG]TTTCTGGGGGGTTGCACATGTATGTGAGGCCACACATATGTGTATGTATGTGTGATTGTG,37,1,3238152,TACATACACATATGTGTGGCCTCACATACATGTGCAACCCCCCAGAAACG,F,PRDM16;PRDM16,NM_022114;NM_199454,Body;Body,chr1:3239915-3240261,N_Shore,,,,,,,,PRDM16,ENST00000512462.1,5'UTR,PRDM16;PRDM16,ENST00000512462.1;ENST00000463591.1,5'UTR;5'UTR,,,chr1:3235625-3249044,6,chr1:3236211-3243833,6,,TRUE,1,3228012,rs188968241;rs537016436;rs34069649;rs373971368;rs544951158;rs35135252,1;13;27;37;39;41,0.001198;0.000200;0.500000;0.007987;0.415535;0.004193, |
|  | 7774563 | − 0.47 | 0.00045 | cg12096707,cg12096707,0007774563,TAATAATCCCAAAACAAAAAATATTAACACCTACCRAACTCAACCTAAAC,,,II,,,AGTCGCCCAGATGGTGGTCCCAGGGCAGGGAGTGTTAGCACCTGCCGGGCTCAGCCTGGG[CG]GCTCAGAGGGCGGGACCTGGAGAAGGAGGGCCGCTCGGCAACCGCTGAGCCGGCGCAAGA,37,1,3073714,CGCCCAGGCTGAGCCCGGCAGGTGCTAACACTCCCTGCCCTGGGACCACC,R,PRDM16;PRDM16,NM_022114;NM_199454,Body;Body,chr1:3071899-3072239,S_Shore,,,,,1:3063486-3063630,,,,,,,,,chr1:3073340-3073850,3,,,,,,TRUE,1,3063574,rs559080898;rs138620333,25;16,0.000200;0.004193, |
|  | 7723551 | − 0.46 | 0.00067 | cg22674798,cg22674798,0007723551,AAACTTTAAAAACCRTCTCTCTAACCAAAAAAACTTATACRAAATCACTC,,,II,,,CCTGCTCACCAGGCACTAGCCTCCTTGGAGGGTGTGGAAGATTCCAAGGTGATCCGGCCC[CG]AGTGATCTCGTACAAGCTTTTCTGGTTAGAGAGACGGTTCCCAAAGCCCAGAGATCTGTG,37,1,3096360,GGCTTTGGGAACCGTCTCTCTAACCAGAAAAGCTTGTACGAGATCACTCG,F,PRDM16;PRDM16,NM_022114;NM_199454,Body;Body,,,,,,,1:3086214-3086455,,,,,,,,,,,chr1:3095056-3096579,6,,,,TRUE,1,3086220,rs532990741;rs187903794,11;33,0.000200;0.000200, |
|  | 15664924 | −0.49 | 0.00024 | cg24612696,cg24612696,0015664924,AAAAACTTTTTACCCRAAACAAAACTTTATAAAAATAACAATAACATCRC,,,II,,,CTCTGAAACAAAGAGGCTTTTTACCCGGAACAAGGCTTTATAAAAATGACAGTGGCATCG[CG]AGTCATTCAGCGCCTGCCCGGGGCGATGGAGGCGGCGAGGAAATACAGGATGCGGGCTCT,37,1,3089891,CGCGATGCCACTGTCATTTTTATAAAGCCTTGTTCCGGGTAAAAAGCCTC,R,PRDM16;PRDM16,NM_022114;NM_199454,Body;Body,,,,,,,1:3079749-3080214,,,,,,,,,,,chr1:3084897-3092440,6,,,,TRUE,1,3079751,rs17390062;rs532630229;rs552300491,44;36;6,0.031949;0.000200;0.000399, |
| LYN | 43783906 | − 0.43 | 0.0014 | cg11332505,cg11332505,0043783906,CAACCCAATAACCATAATAACCTCCAAATCACTACCTACTAAAACCRACC,,,II,,,GGAGAGAGACACAGCCCAGTGGCCATGGTGGCCTCCAGATCACTACCTGCTAAAGCCGGC[CG]TGTGGAGACGTGGGCTGGGCCGCTCCTTCCCTCTGTGGCTCCCTCCTGTGTTCATTGATC,37,1,3294322,CGGCCGGCTTTAGCAGGTAGTGATCTGGAGGCCACCATGGCCACTGGGCT,R,PRDM16;PRDM16,NM_022114;NM_199454,Body;Body,,,,,,,1:3284178-3284304,,,PRDM16,ENST00000512462.1,5'UTR,PRDM16;PRDM16,ENST00000512462.1;ENST00000463591.1,5'UTR;5'UTR,chr1:3294045-3294430,3,,,,,,TRUE,1,3284182,rs560940099;rs372714133;rs375479942,37;26;12,0.000399;0.001198;0.000200, |
|  | 85623345 | 0.41 | 0.0023 | cg00983956,cg00983956,0085623345,AAAAAATCRATTTTCTAAACTCTTACTAACTTAATAACRTAACRTTCAAC,,,II,,,GTGACTCTCAAGGAAAGTCGGTTTTCTGAGCTCTTACTGGCTTAGTAGCGTGGCGTTCAA[CG]CAGAGCATTCTAGGTAATGTAGTTTTCATAGATCCCGAGGTGGGTGCCGGGGACCCTTTG,37,19,58220669,GAAAGTCGGTTTTCTGAGCTCTTACTGGCTTAGTAGCGTGGCGTTCAACG,R,ZNF154;ZNF154;ZNF154,NM_001085384;NR_110974;NR_110975,TSS200;TSS200;TSS200,chr19:58220189-58220517,S_Shore,,,,,19:62911854-62912586,,,ZNF154;ZNF154;ZNF154,ENST00000512439.2;ENST00000451275.1;ENST00000426889.1,TSS200;TSS200;TSS200,ZNF154;ZNF154;ZNF154,ENST00000317656.4;ENST00000451275.1;ENST00000426889.1,TSS200;TSS200;TSS200,chr19:58219905-58220755,3,,,,,,,19,62912481,rs181933446,41,0.000200, |
|  | 89776172 | − 0.43 | 0.0013 | cg23258188,cg23258188,0089776172,ATATATTACCRACRCAACACCAAACCACTTACAAAAATACTACTACATTC,,,II,,,GCACCTGAGCTGTGTGTTACCGGCGCAGCACCAGGCCACTTGCAGAAGTACTGCTGCATT[CG]CCAGCAAGGGCCGCCATCACGATGCGCCTCGCAGACCGCAGGGCTTGAGCAACAGGAACT,37,1,3075846,TGTGTTACCGGCGCAGCACCAGGCCACTTGCAGAAGTACTGCTGCATTCG,R,PRDM16;PRDM16,NM_022114;NM_199454,Body;Body,chr1:3071899-3072239,S_Shelf,,,,,,,,,,,,,,chr1:3075665-3076090,3,,,,,,TRUE,1,3065706,,,, |
|  | 90682536 | 0.52 | 7e−05 | cg05661282,cg05661282,0090682536,CAAAATAACCACTACCATCAAACTCTACAAATAAAACTAAACCAAAAACA,0039652823,CAAAATAACCGCTACCATCAAACTCTACGAATAAAACTAAACCGAAAACG,I,A,Red,AGTTGGCGTCCTCAGAGTGGCCGCTGCCATCAGACTCTGCGGGTAGAGCTGGGCCGGGAG[CG]ACGGGCGACATTGGTAGGGACCCGGGGACAGCGGTCCCTATCCCAGGCCTGACGTGGGTC,37,19,58220370,CGCTCCCGGCCCAGCTCTACCCGCAGAGTCTGATGGCAGCGGCCACTCTG,R,ZNF154;ZNF154,NM_001085384;NM_001085384,5'UTR;1stExon,chr19:58220189-58220517,Island,,,,,19:62911854-62912586,,,ZNF154;ZNF154;ZNF154;ZNF154;ZNF154;ZNF154,ENST00000512439.2;ENST00000451275.1;ENST00000426889.1;ENST00000512439.2;ENST00000426889.1;ENST00000451275.1,1stExon;1stExon;1stExon;5'UTR;5'UTR;3'UTR,ZNF154;ZNF154;ZNF154;ZNF154;ZNF154;ZNF154;ZNF154,ENST00000317656.4;ENST00000451275.1;ENST00000426889.1;ENST00000512439.2;ENST00000426889.1;ENST00000317656.4;ENST00000451275.1,1stExon;1stExon;1stExon;5'UTR;5'UTR;3'UTR;3'UTR,chr19:58219905-58220755,3,,,,,,TRUE,19,62912182,rs10559485;rs369658700,3;1,0.030351;0.000799, |
|  | 81781495 | 0.43 | 0.0016 | cg15746696,cg15746696,0081781495,TATTACCTAAAAAACTACATTACCCAAAAAACTCAACCTCRAATAATAAC,,,II,,,GGACCCATTGTCACGTGCACACAGGAAACGCCTTTATTCTGAGCCCTCAGAGGTCTTCTA[CG]CTATCATTCGAGGCTGAGTTTTCTGGGTAATGTAGTTCCCTAGGCAACAAGGAGGGTAAA,37,19,58400494,CGCTATCATTCGAGGCTGAGTTTTCTGGGTAATGTAGTTCCCTAGGCAAC,F,ZNF814,NM_001144989,TSS200,chr19:58399945-58400276,S_Shore,,,,,19:63091758-63092317,19:58399513-58400623,NonGene_Associated,ZNF814,ENST00000435989.1,TSS200,ZNF814,ENST00000435989.1,TSS200,chr19:58399545-58400615,3,,,,,,TRUE,19,63092306,rs530143271;rs540912247,25;51,0.000200;0.000799, |
|  | 6809506 | 0.54 | 3.2e−05 | cg15759937,cg15759937,0006809506,AAAAACTCCTAAACRCTAAATCCRCACTATAATAACTAAACCCAAAAATC,,,II,,,CGCTGGGCGCCGTCACAGAGCTCCAGAGTAGCCTCTGTGCAGCGGAGGACAACTGCTCCC[CG]ACTTCTGGGTTCAGTCACCACAGTGCGGACCTAGCGCTCAGGAGCCTCTCCTACAAATAA,37,19,58400325,GAGGCTCCTGAGCGCTAGGTCCGCACTGTGGTGACTGAACCCAGAAGTCG,F,ZNF814;ZNF814,NM_001144989;NM_001144989,1stExon;5'UTR,chr19:58399945-58400276,S_Shore,,,,,19:63091758-63092317,19:58399513-58400623,NonGene_Associated,ZNF814;ZNF814,ENST00000435989.1;ENST00000435989.1,1stExon;5'UTR,ZNF814;ZNF814,ENST00000435989.1;ENST00000435989.1,1stExon;5'UTR,chr19:58399545-58400615,3,,,,,,TRUE,19,63092137,rs540447770,43,0.000399, |
|  | 40784534 | 0.47 | 0.00038 | cg22510337,cg22510337,0040784534,TACAAAAAAAACCCCATTTCTAACACCCATAAAATACAATAAAAAAACCA,0074733139,TACGAAAAAAACCCCATTTCTAACACCCGTAAAATACGATAAAAAAACCG,I,A,Red,TTCCATCCTTCTGGCTCAGTTCACTTCCAGGCCTTTGCCCGCGCCAGTCCCTGTACCTGC[CG]GTCTCCCCACCGCATCCCACGGGTGTCAGAAATGGGGCCCCTCCCGCAAGCGCCTCAGTG,37,19,58399967,TGCGGGAGGGGCCCCATTTCTGACACCCGTGGGATGCGGTGGGGAGACCG,F,ZNF814,NM_001144989,Body,chr19:58399945-58400276,Island,,,,,19:63091758-63092317,19:58399513-58400623,NonGene_Associated,,,,,,,chr19:58399545-58400615,3,,,,,,TRUE,19,63091779,rs575261071;rs537821271;rs554271512,8;22;24,0.000200;0.000200;0.000399, |
|  | 47763189 | − 0.42 | 0.002 | cg12149490,cg12149490,0047763189,ACAATTCATAAAAATAATCTACTTAAAACCATTATACTACCTTACCATCC,,,II,,,TCCAACCAGAAGCAATTCATAAAAATGATCTGCTTAAAACCATTGTGCTGCCTTGCCATC[CG]TTGTCAAAGGTGTGAGGGGTGTGTGTGCACATGTGTTCAGTGTGGGGTGTGCGCACATGT,37,1,3062980,CGGATGGCAAGGCAGCACAATGGTTTTAAGCAGATCATTTTTATGAATTG,R,PRDM16;PRDM16,NM_199454;NM_022114,Body;Body,chr1:3059050-3059268,S_Shelf,,,,,,,,,,,,,,,,chr1:3060367-3066903,6,chr1:3062918-3063852,4,,,1,3052840,rs567083445,1,0.000200, |
|  | 43725908 | − 0.42 | 0.0018 | cg05075921,cg05075921,0043725908,ATAAACAAAAATAACCTCACTATCTACATAACTAATAACCCAACCAAAAC,,,II,,,ACGTGCTTCACTCACCCGCCCCACCCGCTCCCGGTCTCTCACTTCCACCCCTGTAGAAAG[CG]CTCTGGCTGGGCTATCAGCTATGCAGACAGTGAGGTCATTCCTGCCCACCGAGGCTCCCG,37,1,3062287,CGCTCTGGCTGGGCTATCAGCTATGCAGACAGTGAGGTCATTCCTGCCCA,F,PRDM16;PRDM16,NM_199454;NM_022114,Body;Body,chr1:3059050-3059268,S_Shelf,,,,,,,,,,,,,,,,chr1:3060367-3066903,6,,,,,1,3052147,rs564981467;rs575276218;rs117130095;rs138160212;rs112309464;rs189438930,0;23;28;40;46;51,0.000200;0.000200;0.024561;0.000399;0.017772;0.000998, |
|  | 73668587 | 0.46 | 0.00053 | cg27049766,cg27049766,0073668587,ACCATTTTAACTTCTCTAAAATATATTCACCRAATCAAAAATAACAAAAC,,,II,,,TAGACGCTTTCGTGCAGGAGGGACGACGACTCCCCTCACGCCTTCGTGGCCCCAACTCGG[CG]CTCTGCTATCTCTGATCCGGTGAACACACCTCAGAGAAGCTAAAATGGCCGCCACGAAGA,37,19,58220516,CCATTTTAGCTTCTCTGAGGTGTGTTCACCGGATCAGAGATAGCAGAGCG,F,ZNF154;ZNF154,NM_001085384;NM_001085384,5'UTR;1stExon,chr19:58220189-58220517,Island,,,,,19:62911854-62912586,,,ZNF154;ZNF154;ZNF154;ZNF154;ZNF154;ZNF154,ENST00000512439.2;ENST00000451275.1;ENST00000426889.1;ENST00000512439.2;ENST00000426889.1;ENST00000451275.1,1stExon;1stExon;1stExon;5'UTR;5'UTR;3'UTR,ZNF154;ZNF154;ZNF154;ZNF154;ZNF154;ZNF154;ZNF154,ENST00000317656.4;ENST00000451275.1;ENST00000426889.1;ENST00000512439.2;ENST00000426889.1;ENST00000317656.4;ENST00000451275.1,1stExon;1stExon;1stExon;5'UTR;5'UTR;3'UTR;3'UTR,chr19:58219905-58220755,3,,,,,,TRUE,19,62912328,rs572379115;rs187039120,9;50,0.000200;0.002596, |
|  | 99694881 | 0.52 | 7.2e−05 | cg03142586,cg03142586,0099694881,AAAAACCAACACATACAAAAATTAAAACACAACTAAACCAAAAAAATTCA,0099749976,AAAAACCGACGCGTACAAAAATTAAAACGCGACTAAACCAAAAAAATTCG,I,A,Red,AGTACAACCCTAGGAGTAACTCCGCCTCATATTCTTCGTTCCCTGCAGAAAACAGCTTTC[CG]AATTCTCCTGGCTCAGTCGCGCCTCAACCTTTGCACGCGCCGGTTCCTCCGCCTGTCACG,37,19,58220080,AGGAACCGGCGCGTGCAAAGGTTGAGGCGCGACTGAGCCAGGAGAATTCG,F,ZNF154,NM_001085384,Body,chr19:58220189-58220517,N_Shore,,,,,19:62911854-62912586,,,ZNF154,ENST00000451275.1,3'UTR,ZNF154;ZNF154,ENST00000317656.4;ENST00000451275.1,3'UTR;3'UTR,chr19:58219905-58220755,3,,,,,,TRUE,19,62911892,rs552434996;rs115903356;rs375066767,21;28;48,0.001198;0.005391;0.001997, |
|  | 7625948 | 0.51 | 0.00011 | cg02280912,cg02280912,0007625948,TCTCCRCTCAAATAATTATAAAACCTTCTATATCCTCAAATCACCTCATC,,,II,,,CGCCGGCGTCCGGGCTGCAGAGCCGTGAACAGGCGCTGCTACCTCGCTGCTTTTGGGTGA[CG]ATGAGGTGACCTGAGGACACAGAAGGCCCCACAATTACCTGAGCGGAGAGCCTCAGCGTA,37,19,58400095,CTCCGCTCAGGTAATTGTGGGGCCTTCTGTGTCCTCAGGTCACCTCATCG,F,ZNF814,NM_001144989,Body,chr19:58399945-58400276,Island,,,,,19:63091758-63092317,19:58399513-58400623,NonGene_Associated,,,,,,,chr19:58399545-58400615,3,,,,,,TRUE,19,63091907,rs75177271;rs375550431,4;23,0.001997;0.000599, |
|  | 50709158 | 0.44 | 0.0011 | cg27112264,cg27112264,0050709158,CAAAAAACRCCTTTATTCTAAACCCTCAAAAATCTTCTACRCTATCATTC,,,II,,,TCACGTGCACACAGGAAACGCCTTTATTCTGAGCCCTCAGAGGTCTTCTACGCTATCATT[CG]AGGCTGAGTTTTCTGGGTAATGTAGTTCCCTAGGCAACAAGGAGGGTAAAGGGCGCAATT,37,19,58400504,AGGAAACGCCTTTATTCTGAGCCCTCAGAGGTCTTCTACGCTATCATTCG,R,ZNF814,NM_001144989,TSS200,chr19:58399945-58400276,S_Shore,,,,,19:63091758-63092317,19:58399513-58400623,NonGene_Associated,ZNF814,ENST00000435989.1,TSS200,ZNF814,ENST00000435989.1,TSS200,chr19:58399545-58400615,3,,,,,,TRUE,19,63092316,rs544418588;rs193293655,43;17,0.000200;0.000200, |
|  | 47611970 | 0.43 | 0.0014 | cg03234186,cg03234186,0047611970,CRAAATCTATAAAAACTACATTACCTAAAATACTCTACRTTAAACRCCAC,,,II,,,TCCTTTCTTTTTGTGACTCTCAAGGAAAGTCGGTTTTCTGAGCTCTTACTGGCTTAGTAG[CG]TGGCGTTCAACGCAGAGCATTCTAGGTAATGTAGTTTTCATAGATCCCGAGGTGGGTGCC,37,19,58220657,GGGATCTATGAAAACTACATTACCTAGAATGCTCTGCGTTGAACGCCACG,F,ZNF154,NM_001085384,TSS200,chr19:58220189-58220517,S_Shore,,,,,19:62911854-62912586,,,ZNF154;ZNF154;ZNF154,ENST00000512439.2;ENST00000451275.1;ENST00000426889.1,TSS200;TSS200;TSS200,ZNF154;ZNF154;ZNF154,ENST00000317656.4;ENST00000451275.1;ENST00000426889.1,TSS200;TSS200;TSS200,chr19:58219905-58220755,3,,,,,,TRUE,19,62912469,rs150322520,46,0.001597, |
|  | 20736918 | − 0.41 | 0.0028 | cg10965478,cg10965478,0020736918,CAATCTTACTAAACTCCAACCCCAAATTAATTTAATAAAAACTCRAACTC,,,II,,,TACCTGAAAACCAGTCTTACTGAGCTCCAACCCCAAATTAATTTGGTGAGAACTCGAACT[CG]GCTATTAACCTTAATTCTGAGAGAAGAATGACACCCCCCTCCCACTGCGAGGGTCCACTG,37,15,31617633,AGTCTTACTGAGCTCCAACCCCAAATTAATTTGGTGAGAACTCGAACTCG,R,KLF13,NM_015995,TSS1500,chr15:31617763-31620908,N_Shore,,,,,,,,KLF13,ENST00000307145.3,TSS1500,KLF13,ENST00000307145.3,TSS1500,chr15:31617600-31618555,3,,,,,,TRUE,15,29404925,rs556452198,2,0.000200, |
|  | 89629166 | − 0.41 | 0.0026 | cg13084877,cg13084877,0089629166,AAAAACCAATCTTACTAAACTCCAACCCCAAATTAATTTAATAAAAACTC,,,II,,,GTCGGTTACCTGAAAACCAGTCTTACTGAGCTCCAACCCCAAATTAATTTGGTGAGAACT[CG]AACTCGGCTATTAACCTTAATTCTGAGAGAAGAATGACACCCCCCTCCCACTGCGAGGGT,37,15,31617627,CGAGTTCTCACCAAATTAATTTGGGGTTGGAGCTCAGTAAGACTGGTTTT,R,KLF13;KLF13,NM_001302461;NM_015995,TSS1500;TSS1500,chr15:31617763-31620908,N_Shore,,,,,,,,KLF13,ENST00000307145.3,TSS1500,KLF13,ENST00000307145.3,TSS1500,chr15:31617600-31618555,3,,,,,,,15,29404919,,,, |
|  | 15664924 | −0.4 | 0.0033 | cg24612696,cg24612696,0015664924,AAAAACTTTTTACCCRAAACAAAACTTTATAAAAATAACAATAACATCRC,,,II,,,CTCTGAAACAAAGAGGCTTTTTACCCGGAACAAGGCTTTATAAAAATGACAGTGGCATCG[CG]AGTCATTCAGCGCCTGCCCGGGGCGATGGAGGCGGCGAGGAAATACAGGATGCGGGCTCT,37,1,3089891,CGCGATGCCACTGTCATTTTTATAAAGCCTTGTTCCGGGTAAAAAGCCTC,R,PRDM16;PRDM16,NM_022114;NM_199454,Body;Body,,,,,,,1:3079749-3080214,,,,,,,,,,,chr1:3084897-3092440,6,,,,TRUE,1,3079751,rs17390062;rs532630229;rs552300491,44;36;6,0.031949;0.000200;0.000399, |
|  | 2764483 | −0.54 | 3.3e−05 | cg16030758,cg16030758,0002764483,CACRAACACCTAAAATACAAAACTAAAACTAACTATACAAATACTTATTC,,,II,,,CCGGCTGGGAGCACGGGCACCTGGGGTGCAGAGCTGGGGCTGGCTGTGCAGGTGCTTGTT[CG]TGTCAAGAAGCAGTTGGACTTCTCTGTGAGATTCTGCAGATCTCCCCTGTGACTGTCAAG,37,15,31653946,ACGGGCACCTGGGGTGCAGAGCTGGGGCTGGCTGTGCAGGTGCTTGTTCG,R,KLF13;KLF13,NM_015995;NM_001302461,Body;Body,,,,chr15:31653483-31653483,,,,15:31653131-31654123,Promoter_Associated,,,,KLF13,ENST00000558921.1,5'UTR,chr15:31653025-31654295,3,,,,,,,15,29441238,rs550518078;rs570613126,49;2,0.001198;0.000200, |
| XPNPEP1 | 53694927 | 0.41 | 0.0027 | cg08812189,cg08812189,0053694927,TAAACRCTTTAAACAAATAACTACCTCCCAACRCTCTAAATTAAAAAACC,,,II,,,AAGAAGGTTTCTGGGCGCTTTAAACAAATGGCTGCCTCCCAGCGCTCTGAGTTAAGGGAC[CG]GCTACCTAGCGTCTAGCTGAGGAGGAAGACGCGCAGCTGGAGAACTGTTGCCTTTGTAGT,37,3,147110367,GGGCGCTTTAAACAAATGGCTGCCTCCCAGCGCTCTGAGTTAAGGGACCG,R,ZIC4;ZIC4;ZIC4;ZIC4;ZIC4,NM_001168378;NR_033118;NR_033119;NM_032153;NM_001168379,Body;TSS200;TSS200;Body;Body,chr3:147108511-147111703,Island,,,,TRUE,3:148591852-148593286,,,ZIC4,ENST00000472749.2,TSS200,ZIC4;ZIC4;ZIC4;ZIC1;ZIC4;ZIC1;ZIC4;ZIC4;ZIC4,ENST00000494569.1;ENST00000464502.1;ENST00000494569.1;ENST00000472523.1;ENST00000472749.2;ENST00000488404.1;ENST00000475502.1;ENST00000463850.1;ENST00000493664.1,3'UTR;3'UTR;1stExon;TSS1500;TSS200;TSS1500;TSS200;TSS200;3'UTR,chr3:147109660-147110775,3,,,,,,TRUE,3,148593057,rs545170605,44,0.000200, |
|  | 42722571 | 0.45 | 0.00084 | cg15105326,cg15105326,0042722571,ATATACTTACACAACAAACTAAAATACATATAACACTTATCACAACCCCA,0091777103,ATATACTTACGCAACGAACTAAAATACGTATAACACTTATCGCAACCCCG,I,C,Grn,CCGTGCACCTTCATGTGCTTACGCAGCGAGCTGGGGTGCGTGTAGCACTTGTCGCAGCCC[CG]CACCTTGCACGTGTATGGCTTGTCGCTAGTGTGCACGTGCGAATGCTTCTTACGGTCGCT,37,3,147108916,CGGGGCTGCGACAAGTGCTACACGCACCCCAGCTCGCTGCGTAAGCACAT,R,ZIC4;ZIC4;ZIC4;ZIC4;ZIC4,NM_001168378;NR_033119;NM_032153;NR_033118;NM_001168379,Body;Body;Body;Body;Body,chr3:147108511-147111703,Island,,,,,3:148591202-148591799,,,ZIC4,ENST00000472749.2,3'UTR,ZIC4;ZIC4;ZIC4;ZIC4;ZIC4;ZIC4;ZIC4,ENST00000472749.2;ENST00000494569.1;ENST00000463850.1;ENST00000464502.1;ENST00000475502.1;ENST00000494569.1;ENST00000493664.1,3'UTR;3'UTR;3'UTR;3'UTR;3'UTR;1stExon;3'UTR,chr3:147108725-147109115,3,,,,,,TRUE,3,148591606,rs75921971,36,0.005591, |
|  | 3664217 | 0.54 | 3.8e−05 | cg23189410,cg23189410,0003664217,CTCAATTAACCTATCTTTACCTAACACAAAATCTATTCAACAACTACACA,0064654282,CTCGATTAACCTATCTTTACCTAACGCAAAATCTATTCAACAACTACGCG,I,T,Red,TTCAACAATCATTTTAATATATAGTCAATGGCTCTTTGTGGAAGGGACAAAAAGAAACTA[CG]CGCAGTTGTTGAATAGACTTTGCGCTAGGCAAAGACAGGTTAATCGAGGGCCGCATCGCG,37,3,147125712,CGCGCAGTTGTTGAATAGACTTTGCGCTAGGCAAAGACAGGTTAATCGAG,F,ZIC4;ZIC1,NM_032153;NM_003412,TSS1500;TSS1500,chr3:147126988-147128999,N_Shore,,,RDMR,TRUE,,,,ZIC4;ZIC4;ZIC1,ENST00000491672.1;ENST00000383075.3;ENST00000282928.4,TSS1500;TSS1500;TSS1500,ZIC4;ZIC4;ZIC4;ZIC4;ZIC1;ZIC1,ENST00000463250.1;ENST00000491672.1;ENST00000464144.1;ENST00000383075.3;ENST00000282928.4;ENST00000472523.1,TSS1500;TSS1500;TSS1500;TSS1500;TSS1500;5'UTR,chr3:147124905-147126215,3,,,,,,TRUE,3,148608402,,,, |
|  | 35779210 | 0.52 | 8.4e−05 | cg17546247,cg17546247,0035779210,ACCCTCRATTAACCTATCTTTACCTAACRCAAAATCTATTCAACAACTAC,,,II,,,CAACAATCATTTTAATATATAGTCAATGGCTCTTTGTGGAAGGGACAAAAAGAAACTACG[CG]CAGTTGTTGAATAGACTTTGCGCTAGGCAAAGACAGGTTAATCGAGGGCCGCATCGCGAA,37,3,147125714,CCCTCGATTAACCTGTCTTTGCCTAGCGCAAAGTCTATTCAACAACTGCG,F,ZIC4;ZIC1,NM_032153;NM_003412,TSS1500;TSS1500,chr3:147126988-147128999,N_Shore,,,RDMR,TRUE,,,,ZIC4;ZIC4;ZIC1,ENST00000491672.1;ENST00000383075.3;ENST00000282928.4,TSS1500;TSS1500;TSS1500,ZIC4;ZIC4;ZIC4;ZIC4;ZIC1;ZIC1,ENST00000463250.1;ENST00000491672.1;ENST00000464144.1;ENST00000383075.3;ENST00000282928.4;ENST00000472523.1,TSS1500;TSS1500;TSS1500;TSS1500;TSS1500;5'UTR,chr3:147124905-147126215,3,,,,,,TRUE,3,148608404,,,, |
|  | 85616592 | 0.41 | 0.0028 | cg03900143,cg03900143,0085616592,TAAAAACAACTATCCACACCAAAAAAATTCACTTTATAACAATCACAACA,0045629949,TAAAAACGACTATCCGCACCGAAAAAATTCACTTTATAACGATCACAACG,I,A,Red,CCAGGCCGAGCGCGGTTGCTGGCCCGCGCCTCCCTCCCCGAGGCACCATTGTTCCGGGAT[CG]CTGTGACCGCCACAAAGTGAATCCTTTCGGTGCGGACAGTCGCCTTCAAAGCCAGGCCCC,37,3,147111660,CGCTGTGACCGCCACAAAGTGAATCCTTTCGGTGCGGACAGTCGCCTTCA,F,ZIC4;ZIC4;ZIC4;ZIC4;ZIC4,NM_001168378;NR_033119;NM_032153;NM_001168379;NR_033118,Body;TSS1500;Body;Body;TSS1500,chr3:147108511-147111703,Island,,,,,3:148593633-148594412,,,ZIC4,ENST00000472749.2,TSS1500,ZIC4;ZIC4;ZIC4;ZIC4;ZIC1;ZIC1;ZIC4;ZIC4;ZIC4;ZIC1,ENST00000464502.1;ENST00000472749.2;ENST00000475502.1;ENST00000463850.1;ENST00000472523.1;ENST00000488404.1;ENST00000464502.1;ENST00000493664.1;ENST00000494569.1;ENST00000472523.1,3'UTR;TSS1500;TSS1500;TSS1500;1stExon;TSS200;1stExon;3'UTR;TSS1500;5'UTR,chr3:147111360-147111875,3,,,,,,TRUE,3,148594350,rs538735167;rs16859414,22;34,0.000200;0.047324, |
|  | 58709844 | 0.46 | 0.00052 | cg16790847,cg16790847,0058709844,TCCTAAAAACTACRACCTACTCCCCAAAAAACTAATTACTACTCTTATTC,,,II,,,AGGGGCCAGAATCCTAGGAGCTGCGGCCTGCTCCCCAGAGAGCTAGTTGCTGCTCTTGTT[CG]CATTGGAGATAAAGAAAGCCAAGTCCCGAACCCACTGCTGGCCGCGCGCAGAGAGCAGAT,37,3,147123429,CCTAGGAGCTGCGGCCTGCTCCCCAGAGAGCTAGTTGCTGCTCTTGTTCG,R,ZIC4;ZIC4;ZIC4,NM_032153;NM_001168378;NM_001168379,5'UTR;TSS1500;TSS200,chr3:147126988-147128999,N_Shelf,,,,,,,,ZIC4;ZIC4;ZIC4;ZIC4,ENST00000491672.1;ENST00000383075.3;ENST00000525172.2;ENST00000425731.3,5'UTR;5'UTR;TSS1500;TSS200,ZIC4;ZIC4;ZIC4;ZIC4;ZIC4;ZIC4;ZIC4;ZIC1,ENST00000462748.2;ENST00000463250.1;ENST00000491672.1;ENST00000383075.3;ENST00000525172.2;ENST00000464144.1;ENST00000425731.3;ENST00000472523.1,5'UTR;5'UTR;5'UTR;5'UTR;TSS1500;3'UTR;TSS200;5'UTR,chr3:147123325-147123735,3,,,,,,TRUE,3,148606119,,,, |
|  | 98731966 | 0.42 | 0.0017 | cg12892506,cg12892506,0098731966,CATACATAATACTAAAAATTTTAAAACAAAAACTCAAAATCACAATACCA,0049639227,CGTACATAATACTAAAAATTTTAAAACAAAAACTCGAAATCGCGATACCG,I,T,Red,GCGTGACCAGCTCGTGCATGGTGCTGAAAGTTTTGGAGCAGAGGCTCGGGGTCGCGGTGC[CG]TCGGCCGCCAGCCACTTGCAGATGAGCTCCTGTTTGATGGGCTGGCGCATGTAGCGGAAG,37,3,147113918,CGGCACCGCGACCCCGAGCCTCTGCTCCAAAACTTTCAGCACCATGCACG,R,ZIC4;ZIC4;ZIC4,NM_001168378;NM_032153;NM_001168379,Body;Body;Body,chr3:147113608-147114479,Island,,,RDMR,TRUE,3:148596274-148597489,,,,,,ZIC4;ZIC4;ZIC1,ENST00000493664.1;ENST00000493664.1;ENST00000472523.1,1stExon;3'UTR;5'UTR,chr3:147113700-147114175,3,,,,,,TRUE,3,148596608,rs535827867;rs200350429,18;15,0.000200;0.001000, |
|  | 77611150 | 0.43 | 0.0013 | cg17003736,cg17003736,0077611150,CCTTTCAAAACTACTAAACCTCTAAAATAAAAATCTAAATCTTTCACAAC,,,II,,,AAGAGGCGGCGTTGGGCTAGGCCCCTGCAGCCCGCTCGGAGCGTCCTAGGCCCGGGGCTG[CG]CTGTGAAAGACCCAGATTCTCATCCCAGAGGCCCAGCAGTCCTGAAAGGCCTCCTCTCCG,37,3,147111308,CTTTCAGGACTGCTGGGCCTCTGGGATGAGAATCTGGGTCTTTCACAGCG,F,ZIC4;ZIC4;ZIC4;ZIC4;ZIC4,NM_001168378;NR_033119;NM_032153;NM_001168379;NR_033118,Body;TSS1500;Body;Body;TSS1500,chr3:147108511-147111703,Island,,,,,3:148593633-148594412,,,ZIC4,ENST00000472749.2,TSS1500,ZIC4;ZIC1;ZIC4;ZIC4;ZIC4;ZIC1;ZIC4;ZIC4;ZIC1,ENST00000464502.1;ENST00000488404.1;ENST00000472749.2;ENST00000475502.1;ENST00000463850.1;ENST00000472523.1;ENST00000493664.1;ENST00000494569.1;ENST00000472523.1,3'UTR;TSS1500;TSS1500;TSS1500;TSS1500;1stExon;3'UTR;TSS1500;5'UTR,chr3:147110960-147111355,3,,,,,,TRUE,3,148593998,rs544535779;rs560827243,9;50,0.000200;0.000200, |
|  | 8602292 | 0.51 | 0.00013 | cg18930354,cg18930354,0008602292,AAATCTCTTCTAAAATAAATCATTACTCAAAATACCTCTCCCTACAACAC,,,II,,,AGCGATGAAATAATTTAAGGATGCGCAGCCGATGCACATTGTGTGTGCATAAAGTGGATT[CG]TGCTGCAGGGAGAGGTATTCTGAGCAATGATTCACTTCAGAAGAGATTTTTACAGGAATG,37,3,147109629,CGTGCTGCAGGGAGAGGTATTCTGAGCAATGATTCACTTCAGAAGAGATT,F,ZIC4;ZIC4;ZIC4;ZIC4;ZIC4,NM_001168378;NR_033119;NM_032153;NR_033118;NM_001168379,Body;Body;Body;Body;Body,chr3:147108511-147111703,Island,,,,,3:148591852-148593286,,,ZIC4,ENST00000472749.2,3'UTR,ZIC4;ZIC4;ZIC4;ZIC4;ZIC4;ZIC4;ZIC4,ENST00000472749.2;ENST00000494569.1;ENST00000463850.1;ENST00000464502.1;ENST00000475502.1;ENST00000494569.1;ENST00000493664.1,3'UTR;3'UTR;3'UTR;3'UTR;3'UTR;1stExon;3'UTR,,,chr3:147108278-147112699,6,chr3:147104865-147116795,6,,TRUE,3,148592319,rs545334995,1,0.000200, |
|  | 36628967 | 0.43 | 0.0014 | cg22203776,cg22203776,0036628967,AAAAAAAACCCTATCATTAAAAATAAATTCCTTCTCCAACTCAAAACTAC,,,II,,,AAAAAAAAAAAAAAAAAAACCCTGTCATTAAAGATGAGTTCCTTCTCCAGCTCAGGACTG[CG]AGTCTACCTTCCGGGTGTCCGCCGCCAGCTCCCGGAGTAGCAGGCCTGGGGCCAGGATTC,37,3,147112316,CGCAGTCCTGAGCTGGAGAAGGAACTCATCTTTAATGACAGGGTTTTTTT,R,ZIC4;ZIC4;ZIC4,NM_001168378;NM_032153;NM_001168379,Body;Body;Body,chr3:147113608-147114479,N_Shore,,,RDMR,,3:148595006-148595248,,,,,,ZIC4;ZIC4;ZIC1,ENST00000493664.1;ENST00000464502.1;ENST00000472523.1,3'UTR;TSS1500;5'UTR,chr3:147112060-147112655,3,,,,,,TRUE,3,148595006,rs9811502,43,0.037500, |
|  | 9791841 | 0.46 | 6e−04 | cg20939084,cg20939084,0009791841,TCTCCAAAAAACCTCTAAAACAACRTAATAAAATATTATTACTAACRAAC,,,II,,,GGCAAACATTTAGCAGCATTCTTCAAATCTTGCCTAAACCTTCCGGGATCCCTCCAGATA[CG]CTCGCCAGTAATAATATTTCATTACGCTGCTCCAGAGGCTTCCTGGAGACCGTGCTGTGG,37,3,147109784,CGCTCGCCAGTAATAATATTTCATTACGCTGCTCCAGAGGCTTCCTGGAG,F,ZIC4;ZIC4;ZIC4;ZIC4;ZIC4,NM_001168378;NR_033119;NM_032153;NR_033118;NM_001168379,Body;Body;Body;Body;Body,chr3:147108511-147111703,Island,,,,,3:148591852-148593286,,,ZIC4;ZIC4,ENST00000472749.2;ENST00000472749.2,3'UTR;1stExon,ZIC4;ZIC4;ZIC4;ZIC4;ZIC4;ZIC4;ZIC1;ZIC4;ZIC4,ENST00000472749.2;ENST00000494569.1;ENST00000463850.1;ENST00000464502.1;ENST00000475502.1;ENST00000494569.1;ENST00000472523.1;ENST00000472749.2;ENST00000493664.1,3'UTR;3'UTR;3'UTR;3'UTR;3'UTR;1stExon;TSS1500;1stExon;3'UTR,chr3:147109660-147110775,3,,,,,,TRUE,3,148592474,rs529678587;rs58262807,35;45,0.001597;0.392772, |
|  | 86661913 | 0.44 | 0.00099 | cg00235367,cg00235367,0086661913,TAACCRATCCCTTAACTCAAAACRCTAAAAAACAACCATTTATTTAAAAC,,,II,,,AAGCCTAGATTCCTGCCGGAGCTGCAAGTGCTGCGGAAATGGGGGAAGAAGGTTTCTGGG[CG]CTTTAAACAAATGGCTGCCTCCCAGCGCTCTGAGTTAAGGGACCGGCTACCTAGCGTCTA,37,3,147110322,AGCCGGTCCCTTAACTCAGAGCGCTGGGAGGCAGCCATTTGTTTAAAGCG,F,ZIC4;ZIC4;ZIC4;ZIC4;ZIC4,NM_001168378;NR_033118;NR_033119;NM_032153;NM_001168379,Body;TSS200;TSS200;Body;Body,chr3:147108511-147111703,Island,,,,TRUE,3:148591852-148593286,,,ZIC4,ENST00000472749.2,TSS200,ZIC4;ZIC4;ZIC4;ZIC1;ZIC4;ZIC1;ZIC4;ZIC4;ZIC4,ENST00000494569.1;ENST00000464502.1;ENST00000494569.1;ENST00000472523.1;ENST00000472749.2;ENST00000488404.1;ENST00000475502.1;ENST00000463850.1;ENST00000493664.1,3'UTR;3'UTR;1stExon;TSS1500;TSS200;TSS1500;TSS200;TSS200;3'UTR,chr3:147109660-147110775,3,,,,,,TRUE,3,148593012,rs545170605,3,0.000200, |
|  | 34662265 | 0.44 | 0.001 | cg19516404,cg19516404,0034662265,CACCCTAATCCTACCTCAAAAAACTAACTACTAAAATCTACTCTCTACRC,,,II,,,TTGCTGCTCTTGTTCGCATTGGAGATAAAGAAAGCCAAGTCCCGAACCCACTGCTGGCCG[CG]CGCAGAGAGCAGATCCCAGCAGTCAGCTTCCTGAGGCAGGACCAGGGTGAGGGAGGGCAA,37,3,147123475,ACCCTGGTCCTGCCTCAGGAAGCTGACTGCTGGGATCTGCTCTCTGCGCG,F,ZIC4;ZIC4;ZIC4,NM_032153;NM_001168378;NM_001168379,5'UTR;TSS1500;TSS200,chr3:147126988-147128999,N_Shelf,,,,,,,,ZIC4;ZIC4;ZIC4;ZIC4,ENST00000491672.1;ENST00000383075.3;ENST00000525172.2;ENST00000425731.3,5'UTR;5'UTR;TSS1500;TSS200,ZIC4;ZIC4;ZIC4;ZIC4;ZIC4;ZIC4;ZIC4;ZIC4;ZIC1,ENST00000462748.2;ENST00000463250.1;ENST00000491672.1;ENST00000383075.3;ENST00000525172.2;ENST00000464144.1;ENST00000425731.3;ENST00000463250.1;ENST00000472523.1,5'UTR;5'UTR;5'UTR;5'UTR;TSS1500;3'UTR;TSS200;ExonBnd;5'UTR,chr3:147123325-147123735,3,,,,,,TRUE,3,148606165,rs184839225;rs543662244,5;10,0.000200;0.000399, |
|  | 48616842 | 0.44 | 0.00098 | cg18082337,cg18082337,0048616842,CTTCAAAACCAAATACAAACTTATAAATCACATCCACATACACACAAACA,0019783576,CTTCAAAACCAAATACAAACTTATAAATCACATCCGCGTACACACGAACG,I,A,Red,GATTTTCTGATCTAGCAAAGACCTTCCCACACCCCGGGAAAGGACAAGGGAAGGGCTTCT[CG]CCCGTGTGCACGCGGATGTGATTTACAAGTTTGTATTTGGCTTTGAAGGGCTTTCCCTGG,37,3,147113726,CGCCCGTGTGCACGCGGATGTGATTTACAAGTTTGTATTTGGCTTTGAAG,F,ZIC4;ZIC4;ZIC4,NM_001168378;NM_032153;NM_001168379,Body;Body;Body,chr3:147113608-147114479,Island,,,RDMR,TRUE,3:148596274-148597489,,,,,,ZIC4;ZIC4;ZIC1,ENST00000493664.1;ENST00000493664.1;ENST00000472523.1,1stExon;3'UTR;5'UTR,chr3:147113700-147114175,3,,,,,,TRUE,3,148596416,rs75011808,25,0.000399, |
|  | 92688890 | 0.45 | 9e−04 | cg12388007,cg12388007,0092688890,AAAAAACAAACRCTTTAAAACTAATTAAAACAAACAAAACCCCAATTCCC,,,II,,,GCCGCATCCAGGAAAAACAGGCGCTTTGGGGCTGGTTAGAACAAACAAAGCCCCAATTCC[CG]AGCCCTGTTGAGGCTCGGACAGAGAGGTTTGCGCACAACCTGCGCTTCTGCGCAATCAGC,37,3,147110499,CGGGAATTGGGGCTTTGTTTGTTCTAACCAGCCCCAAAGCGCCTGTTTTT,R,ZIC4;ZIC4;ZIC4;ZIC4;ZIC4,NM_001168378;NR_033119;NM_032153;NM_001168379;NR_033118,Body;TSS1500;Body;Body;TSS1500,chr3:147108511-147111703,Island,,,DMR,TRUE,3:148591852-148593286,,,ZIC4,ENST00000472749.2,TSS1500,ZIC4;ZIC4;ZIC4;ZIC1;ZIC1;ZIC4;ZIC4;ZIC4;ZIC4,ENST00000494569.1;ENST00000464502.1;ENST00000494569.1;ENST00000472523.1;ENST00000488404.1;ENST00000472749.2;ENST00000475502.1;ENST00000463850.1;ENST00000493664.1,3'UTR;3'UTR;1stExon;TSS1500;TSS1500;TSS1500;TSS1500;TSS1500;3'UTR,chr3:147109660-147110775,3,,,,,,TRUE,3,148593189,rs550553553;rs560955876;rs530034534,37;12;7,0.000200;0.000399;0.000200, |
| GP1BA | 54742598 | − 0.52 | 7.7e−05 | cg10493186,cg10493186,0054742598,CAACCTAACATAACAACCAAAAAAAATACCTAACTCTCAACAAAAAACCA,0002783984,CAACCTAACGTAACGACCAAAAAAAATACCTAACTCTCGACAAAAAACCG,I,C,Grn,AAAGGGCCTCTGCAGCCTGGCGTGACGGCCAGAGGAGGTGCCTGGCTCTCGGCAGGGAGC[CG]CAGGGCTCTTTCTTCCTCTCTGGTTCGTACTTCTCCTCTCTCATGGGGGTCCCCCAAAGC,37,1,3134756,CGGCTCCCTGCCGAGAGCCAGGCACCTCCTCTGGCCGTCACGCCAGGCTG,R,PRDM16;PRDM16,NM_022114;NM_199454,Body;Body,,,,,,,1:3123982-3124644,,,,,,,,,chr1:3134685-3135075,3,,,,,,TRUE,1,3124616,rs575413405;rs370514834;rs145645797,37;12;10,0.000200;0.000599;0.000599, |
|  | 3787232 | − 0.43 | 0.0015 | cg26425711,cg26425711,0003787232,ACCAAATTCCCCCCTATAACTTCAAAAAAATAAACRCCCTAAAAAAACTC,,,II,,,GTGGCAGGGAGACCAGGTTCCCCCCTGTGGCTTCAGGAGGGTGGGCGCCCTGAGAGAACT[CG]CTTCAAGGCCCCTGTCCCTGCCTGGAAGGAGCCCAGCGTGCGAATTCCAGCTGCCCTGCA,37,1,3251680,CCAGGTTCCCCCCTGTGGCTTCAGGAGGGTGGGCGCCCTGAGAGAACTCG,R,PRDM16;PRDM16,NM_022114;NM_199454,Body;Body,,,,,,,1:3241339-3241889,,,PRDM16,ENST00000512462.1,5'UTR,PRDM16;PRDM16,ENST00000512462.1;ENST00000463591.1,5'UTR;5'UTR,chr1:3251565-3252295,3,,,,,,TRUE,1,3241540,rs560315508;rs532848837;rs552220666;rs569033023;rs553936239,39;26;13;7;1,0.000200;0.000399;0.000200;0.000799;0.001198, |
|  | 88804205 | − 0.44 | 0.00097 | cg17445936,cg17445936,0088804205,CCCTAATAAAATAAATAACAATCACACATCCTCCAAACTATCATAAAACC,,,II,,,TCAGGGCCGGCCCCTGGTGGGGTGAATGGCAGTCACACATCCTCCAGGCTGTCATGGGGC[CG]AGGCTCCGTGCTCAGCAATGCGGTTCCTCTGGGTGGTTTATGCTCCCGGTGCAAAGCCTT,37,1,3090345,CGGCCCCATGACAGCCTGGAGGATGTGTGACTGCCATTCACCCCACCAGG,R,PRDM16;PRDM16,NM_022114;NM_199454,Body;Body,,,,,,,1:3079749-3080214,,,,,,,,,chr1:3090200-3090475,3,,,,,,TRUE,1,3080205,rs114106344;rs115133463,10;2,0.046725;0.001797, |
|  | 97777585 | − 0.4 | 0.0029 | cg22510139,cg22510139,0097777585,ACCAAATATTTTCACTCRATTTTACAAATCTACTCCAAATCTAAAACATC,,,II,,,AGCTGTCAGTCAAGCGTCTTTCACAGCCAGCCAAGACCTTTTGATTTCTAGAACAAACAG[CG]ATGTCTTAGACCTGGAGCAGATTTGCAAAATCGAGTGAAAACATCTGGCTGTGCTAACAA,37,1,3058822,CCAGATGTTTTCACTCGATTTTGCAAATCTGCTCCAGGTCTAAGACATCG,F,PRDM16;PRDM16,NM_022114;NM_199454,Body;Body,chr1:3059050-3059268,N_Shore,,,,,1:3048260-3049151,,,,,,,,,,,chr1:3049249-3060070,6,,,,TRUE,1,3048682,,,, |
|  | 99772122 | − 0.46 | 6e−04 | cg12473797,cg12473797,0099772122,ACTACTAAACAAAAAACAATCCRACRTAACAAAAACAAACCTTTCTCAAC,,,II,,,TATGACTGATCCTTTTTCTTCACGTAAGCGGTGGAGTTAAGTGTTTGGAATTGCTGCAGA[CG]CTGAGAAAGGCCTGCCCCTGTCACGCCGGATTGTCCCCTGCTCAGCAGCTGACACAGCCC,37,1,3036358,CGCTGAGAAAGGCCTGCCCCTGTCACGCCGGATTGTCCCCTGCTCAGCAG,F,PRDM16;PRDM16,NM_022114;NM_199454,Body;Body,chr1:3038067-3038343,N_Shore,,,,,,1:3036332-3036547,Unclassified_Cell_type_specific,,,,,,,chr1:3036340-3036655,3,,,,,,TRUE,1,3026218,rs141049593;rs545903303;rs113911649,0;29;39,0.001597;0.000200;0.006789, |
|  | 12656295 | − 0.43 | 0.0015 | cg01431482,cg01431482,0012656295,AATAAAAAAACTACAACCCCCACTAAATAAAAACTATATATATCTTACTC,,,II,,,CTAGCCCCTGGGGTGGGGGAGCTGCAGCCCCCACTAGATGGGGACTGTGTGTGTCTTGCT[CG]TGTTTGAGGGTGATGCGTGTGGAAGGTGTGCTGGAGACAGGGTAGAGGTAAGCGGGGCTG,37,1,2989085,CGAGCAAGACACACACAGTCCCCATCTAGTGGGGGCTGCAGCTCCCCCAC,R,PRDM16;PRDM16,NM_022114;NM_199454,Body;Body,chr1:2990030-2990718,N_Shore,,,,,,,,,,,,,,chr1:2988825-2989375,3,,,,,,TRUE,1,2978945,rs546014913,29,0.000799, |
|  | 70796586 | − 0.4 | 0.0031 | cg11946666,cg11946666,0070796586,AAATAAACCTAAATACRAAAAATAACAACAATTCCATCTAATAAAACCCC,,,II,,,CAGCTCACAGGAAGTGGGCCTGAATGCGGAGAATAGCAGCAATTCCATCTGATAAGGCCC[CG]CTAAACCCCACATTGACAGGCCCCACCGCCCACCCGGGCTCCAGGGGCCAAGGGAGGGGC,37,1,3247595,CGGGGCCTTATCAGATGGAATTGCTGCTATTCTCCGCATTCAGGCCCACT,R,PRDM16;PRDM16,NM_022114;NM_199454,Body;Body,,,,,,,,1:3247537-3247655,Unclassified_Cell_type_specific,PRDM16,ENST00000512462.1,5'UTR,PRDM16;PRDM16,ENST00000512462.1;ENST00000463591.1,5'UTR;5'UTR,chr1:3247425-3247715,3,,,,,,TRUE,1,3237455,rs535391853;rs76122673,44;25,0.000200;0.085064, |
|  | 63733589 | − 0.44 | 0.0012 | cg25618424,cg25618424,0063733589,CCAAAACCAAAACRACAACCTTCCCTCTAAAAACCRATTAAAACAACAAC,,,II,,,CAGTCCCCTCCCCAGAGCCAGGACGGCAGCCTTCCCTCTGAAGGCCGGTTAGGGCAGCAG[CG]CCAGGACGTCCGGCGGGAATCCAGCGTCTTGCCCCGCTTCGGACGAAAACACAGAGGCTG,37,1,2989307,CAGAGCCAGGACGGCAGCCTTCCCTCTGAAGGCCGGTTAGGGCAGCAGCG,R,PRDM16;PRDM16,NM_022114;NM_199454,Body;Body,chr1:2990030-2990718,N_Shore,,chr1:2989176-2989176,,,1:2979167-2979278,,,,,,,,,chr1:2988825-2989375,3,,,,,,TRUE,1,2979167,rs146758878;rs527703576;rs547776195;rs372464526;rs532428462,38;29;15;14;5,0.011582;0.000399;0.000599;0.002995;0.000200, |

**Table S3. The summary of correlation analyses between proteomics data and methylation signatures of transcription factors in TF2DNA database.**

| Proteomic candidates  from GSEA analyses | Methylation probe  in EPIC arry | Pearson coefficient | Correlation  P value | EPIC array info  (IlmnID, Name, AddressA_ID, AlleleA_ProbeSeq, AddressB_ID, AlleleB_ProbeSeq, Infinium_Design_Type, Next_Base, Color_Channel, Forward_Sequence, Genome_Build, CHR, MAPINFO, SourceSeq, Strand, UCSC_RefGene_Name, UCSC_RefGene_Accession, UCSC_RefGene_Group, UCSC_CpG_Islands_Name, Relation_to_UCSC_CpG_Island, Phantom4_Enhancers, Phantom5_Enhancers, DMR, 450k_Enhancer, HMM_Island, Regulatory_Feature_Name, Regulatory_Feature_Group, GencodeBasicV12_NAME, GencodeBasicV12_Accession, GencodeBasicV12_Group, GencodeCompV12_NAME, GencodeCompV12_Accession, GencodeCompV12_Group, DNase_Hypersensitivity_NAME, DNase_Hypersensitivity_Evidence_Count, OpenChromatin_NAME, OpenChromatin_Evidence_Count, TFBS_NAME, TFBS_Evidence_Count, Methyl27_Loci, Methyl450_Loci, Chromosome_36, Coordinate_36, SNP_ID, SNP_DISTANCE, SNP_MinorAlleleFrequency, Random_Loci) |
| --- | --- | --- | --- | --- |
| FYN | 82707370 | − 0.48 | 0.00028 | cg24741666,cg24741666,0082707370,CTATAAAAATCAAACRAACACRTCCAACCATAACCTAAACACAAACAACC,,,II,,,GCGCACGGTGTCTGTGGAGGTCAGGCGGACACGTCCAGCCATGGCCTGGACACAGACAGC[CG]GGCTGTGTGGCACAGGACTGCCTGCTGGAAGCTTCCGCCTGCGCACTCCTCCCGGCCTCC,37,1,3038707,TGTGGAGGTCAGGCGGACACGTCCAGCCATGGCCTGGACACAGACAGCCG,R,PRDM16;PRDM16,NM_022114;NM_199454,Body;Body,chr1:3038067-3038343,S_Shore,,,,,1:3027842-3028807,,,,,,,,,,,chr1:3030640-3044810,6,,,,TRUE,1,3028567,,,, |
|  | 98652496 | − 0.45 | 0.00073 | cg10442735,cg10442735,0098652496,AATACCCACRTATACTATATATAAAAATATATCTAAATATTTTCCTACTC,,,II,,,CTCCTGGGCCCCGTTAGGGACGCAATGAGGACCCGTTTTCTGCGGGACAGAGGGTTTGGC[CG]AGCAGGAAAACATTTAGACACACCCCCACACACAGCACACGTGGGCATCAGGGCTCTGCT,37,1,3062633,ATGCCCACGTGTGCTGTGTGTGGGGGTGTGTCTAAATGTTTTCCTGCTCG,F,PRDM16;PRDM16,NM_022114;NM_199454,Body;Body,chr1:3059050-3059268,S_Shelf,,,,,1:3052292-3052708,,,,,,,,,,,chr1:3060367-3066903,6,,,,TRUE,1,3052493,rs377194829;rs180885402;rs530307548,24;41;42,0.000200;0.000799;0.000599, |
|  | 10660204 | − 0.47 | 0.00047 | cg09335331,cg09335331,0010660204,AATATTCTTAATCATTCTCTCTCCTTTTTTCTAAAATTCCTTATATACCC,,,II,,,ACGTTTTTAGCCCAAGAATGCCATGAAATCCTGCACTATCTTCGGCCCGGCTCGGAGGCA[CG]GGTATATAAGGAATTTTAGAAAAAAGGAGAGAGAATGACTAAGAATACCTCCCTGATGGC,37,1,3111908,CGGGTATATAAGGAATTTTAGAAAAAAGGAGAGAGAATGACTAAGAATAC,F,PRDM16;PRDM16,NM_199454;NM_022114,Body;Body,chr1:3111579-3111909,Island,,chr1:3111769-3111769,,,1:3101029-3101769,,,,,,,,,chr1:3111765-3111995,3,,,,,,,1,3101768,,,, |
|  | 60743243 | − 0.47 | 5e−04 | cg05641535,cg05641535,0060743243,TCRAATTCTTACCCAAATAAAAAACCCTAAAAACTACTCAATTAATCCTC,,,II,,,ACTAAACGCTCCTGGGAGGCCCTAGTGAAGCATCACAGACCATTAGACCGGGGCTTCCGC[CG]AGGACTAATTGAGCAGCCCTTAGGGCTTTTCATCTGGGCAAGAATCCGAACTCCTCCAGG,37,1,3026154,CGGATTCTTGCCCAGATGAAAAGCCCTAAGGGCTGCTCAATTAGTCCTCG,F,PRDM16;PRDM16,NM_022114;NM_199454,Body;Body,chr1:3028179-3028557,N_Shelf,,,,,,1:3026046-3026158,Unclassified_Cell_type_specific,,,,,,,chr1:3025905-3026415,3,,,,,,TRUE,1,3016014,rs572490825;rs545337043,26;45,0.000200;0.001198, |
|  | 90782987 | − 0.47 | 0.00048 | cg03844268,cg03844268,0090782987,CTATTTACCACAATAACTATACCATTTCACATCCCRAAATACCTATATTC,,,II,,,GAACCTCCAGACTGTTTACCACAGTGGCTATACCATTTCACATCCCGGGGTGCCTGTGTT[CG]TTTGACTCAGAGCTGCTCAGAAACGAGGAGCGGCCTCACAGAGCGGCTGAAGGGGCGGCC,37,1,3184187,CGAACACAGGCACCCCGGGATGTGAAATGGTATAGCCACTGTGGTAAACA,R,PRDM16;PRDM16,NM_199454;NM_022114,Body;Body,chr1:3182821-3183216,S_Shore,,,,,1:3173758-3174684,,,PRDM16,ENST00000512462.1,5'UTR,PRDM16;PRDM16,ENST00000512462.1;ENST00000463591.1,5'UTR;5'UTR,chr1:3184045-3184255,3,,,,,,,1,3174047,rs182289293;rs564678747;rs137978473,40;35;30,0.001797;0.000200;0.001597, |
|  | 90774563 | − 0.49 | 0.00027 | cg14030836,cg14030836,0090774563,TAAATTCCCTCACAACAAACTCAAAAAACCRCTTTACCAAACAAAACCTC,,,II,,,ACGCGTGGGGTGCCCACTTCAAATGTGCAGAACCAGGAGCAGCCTCTGCTCTGCCCACCT[CG]AGGCCCTGCTTGGCAAAGCGGCTTTCTGAGCCTGCTGTGAGGGAACCCACCAGCCCCGCG,37,1,2992620,CGAGGCCCTGCTTGGCAAAGCGGCTTTCTGAGCCTGCTGTGAGGGAACCC,F,PRDM16;PRDM16,NM_022114;NM_199454,Body;Body,chr1:2990030-2990718,S_Shore,,,,,1:2982111-2982672,1:2992545-2992737,Unclassified_Cell_type_specific,,,,,,,chr1:2992600-2992975,3,,,,,,TRUE,1,2982480,rs180711049,51,0.000200, |
|  | 81665904 | − 0.48 | 0.00028 | cg03157741,cg03157741,0081665904,CAAAAAAATATCCTAACTCAAAATAACAAACAAAAACCCACCCRTAAACC,,,II,,,AATGCAGTCACCAGGGAGGTGTCCTGGCTCAGGATAGCAGACAGGGACCCACCCGTAAAC[CG]TGTTGTGCTGCTTTAAATTCAGCAGGATTTTTTCTTCTTTTCCCATGAGTTTTTATTCAC,37,1,3285226,CGGTTTACGGGTGGGTCCCTGTCTGCTATCCTGAGCCAGGACACCTCCCT,R,PRDM16;PRDM16,NM_199454;NM_022114,Body;Body,,,,,,,,,,PRDM16,ENST00000512462.1,5'UTR,PRDM16;PRDM16,ENST00000512462.1;ENST00000463591.1,5'UTR;5'UTR,chr1:3285165-3285350,3,,,,,,,1,3275086,rs541699597,8,0.000200, |
|  | 50741930 | − 0.46 | 0.00069 | cg11727198,cg11727198,0050741930,AACAATAACATACATACRTATCTAAACACATATACACACAAACACAAATC,,,II,,,ACATGTGTGCAAACAGTGACATGCATGCGTGTCTAGGCACATGTACACACAAACACAGGT[CG]TGCATGCAAAACACATGTGCACACAGATGTCCACACACATATACAATTTCCTCATGGAGA,37,1,3138369,CGACCTGTGTTTGTGTGTACATGTGCCTAGACACGCATGCATGTCACTGT,R,PRDM16;PRDM16,NM_199454;NM_022114,Body;Body,,,,,,,,,,,,,,,,,,,,chr1:3138216-3139274,6,,,1,3128229,rs557124305;rs116020406,35;1,0.000200;0.004593, |
|  | 47763189 | − 0.46 | 0.00069 | cg12149490,cg12149490,0047763189,ACAATTCATAAAAATAATCTACTTAAAACCATTATACTACCTTACCATCC,,,II,,,TCCAACCAGAAGCAATTCATAAAAATGATCTGCTTAAAACCATTGTGCTGCCTTGCCATC[CG]TTGTCAAAGGTGTGAGGGGTGTGTGTGCACATGTGTTCAGTGTGGGGTGTGCGCACATGT,37,1,3062980,CGGATGGCAAGGCAGCACAATGGTTTTAAGCAGATCATTTTTATGAATTG,R,PRDM16;PRDM16,NM_199454;NM_022114,Body;Body,chr1:3059050-3059268,S_Shelf,,,,,,,,,,,,,,,,chr1:3060367-3066903,6,chr1:3062918-3063852,4,,,1,3052840,rs567083445,1,0.000200, |
|  | 59788837 | − 0.48 | 0.00035 | cg05573412,cg05573412,0059788837,ATACATACACATATATATAACCTCACATACATATACAACCCCCCAAAAAC,,,II,,,GTGTGTGTGTATGTACGTTTGTGTGTGAGTGCAAATGTGTGTGAGAGTGTTTGGAGGTCA[CG]TTTCTGGGGGGTTGCACATGTATGTGAGGCCACACATATGTGTATGTATGTGTGATTGTG,37,1,3238152,TACATACACATATGTGTGGCCTCACATACATGTGCAACCCCCCAGAAACG,F,PRDM16;PRDM16,NM_022114;NM_199454,Body;Body,chr1:3239915-3240261,N_Shore,,,,,,,,PRDM16,ENST00000512462.1,5'UTR,PRDM16;PRDM16,ENST00000512462.1;ENST00000463591.1,5'UTR;5'UTR,,,chr1:3235625-3249044,6,chr1:3236211-3243833,6,,TRUE,1,3228012,rs188968241;rs537016436;rs34069649;rs373971368;rs544951158;rs35135252,1;13;27;37;39;41,0.001198;0.000200;0.500000;0.007987;0.415535;0.004193, |
|  | 7774563 | − 0.47 | 0.00045 | cg12096707,cg12096707,0007774563,TAATAATCCCAAAACAAAAAATATTAACACCTACCRAACTCAACCTAAAC,,,II,,,AGTCGCCCAGATGGTGGTCCCAGGGCAGGGAGTGTTAGCACCTGCCGGGCTCAGCCTGGG[CG]GCTCAGAGGGCGGGACCTGGAGAAGGAGGGCCGCTCGGCAACCGCTGAGCCGGCGCAAGA,37,1,3073714,CGCCCAGGCTGAGCCCGGCAGGTGCTAACACTCCCTGCCCTGGGACCACC,R,PRDM16;PRDM16,NM_022114;NM_199454,Body;Body,chr1:3071899-3072239,S_Shore,,,,,1:3063486-3063630,,,,,,,,,chr1:3073340-3073850,3,,,,,,TRUE,1,3063574,rs559080898;rs138620333,25;16,0.000200;0.004193, |
|  | 7723551 | − 0.46 | 0.00067 | cg22674798,cg22674798,0007723551,AAACTTTAAAAACCRTCTCTCTAACCAAAAAAACTTATACRAAATCACTC,,,II,,,CCTGCTCACCAGGCACTAGCCTCCTTGGAGGGTGTGGAAGATTCCAAGGTGATCCGGCCC[CG]AGTGATCTCGTACAAGCTTTTCTGGTTAGAGAGACGGTTCCCAAAGCCCAGAGATCTGTG,37,1,3096360,GGCTTTGGGAACCGTCTCTCTAACCAGAAAAGCTTGTACGAGATCACTCG,F,PRDM16;PRDM16,NM_022114;NM_199454,Body;Body,,,,,,,1:3086214-3086455,,,,,,,,,,,chr1:3095056-3096579,6,,,,TRUE,1,3086220,rs532990741;rs187903794,11;33,0.000200;0.000200, |
|  | 15664924 | −0.49 | 0.00024 | cg24612696,cg24612696,0015664924,AAAAACTTTTTACCCRAAACAAAACTTTATAAAAATAACAATAACATCRC,,,II,,,CTCTGAAACAAAGAGGCTTTTTACCCGGAACAAGGCTTTATAAAAATGACAGTGGCATCG[CG]AGTCATTCAGCGCCTGCCCGGGGCGATGGAGGCGGCGAGGAAATACAGGATGCGGGCTCT,37,1,3089891,CGCGATGCCACTGTCATTTTTATAAAGCCTTGTTCCGGGTAAAAAGCCTC,R,PRDM16;PRDM16,NM_022114;NM_199454,Body;Body,,,,,,,1:3079749-3080214,,,,,,,,,,,chr1:3084897-3092440,6,,,,TRUE,1,3079751,rs17390062;rs532630229;rs552300491,44;36;6,0.031949;0.000200;0.000399, |
| LYN | 43783906 | − 0.43 | 0.0014 | cg11332505,cg11332505,0043783906,CAACCCAATAACCATAATAACCTCCAAATCACTACCTACTAAAACCRACC,,,II,,,GGAGAGAGACACAGCCCAGTGGCCATGGTGGCCTCCAGATCACTACCTGCTAAAGCCGGC[CG]TGTGGAGACGTGGGCTGGGCCGCTCCTTCCCTCTGTGGCTCCCTCCTGTGTTCATTGATC,37,1,3294322,CGGCCGGCTTTAGCAGGTAGTGATCTGGAGGCCACCATGGCCACTGGGCT,R,PRDM16;PRDM16,NM_022114;NM_199454,Body;Body,,,,,,,1:3284178-3284304,,,PRDM16,ENST00000512462.1,5'UTR,PRDM16;PRDM16,ENST00000512462.1;ENST00000463591.1,5'UTR;5'UTR,chr1:3294045-3294430,3,,,,,,TRUE,1,3284182,rs560940099;rs372714133;rs375479942,37;26;12,0.000399;0.001198;0.000200, |
|  | 85623345 | 0.41 | 0.0023 | cg00983956,cg00983956,0085623345,AAAAAATCRATTTTCTAAACTCTTACTAACTTAATAACRTAACRTTCAAC,,,II,,,GTGACTCTCAAGGAAAGTCGGTTTTCTGAGCTCTTACTGGCTTAGTAGCGTGGCGTTCAA[CG]CAGAGCATTCTAGGTAATGTAGTTTTCATAGATCCCGAGGTGGGTGCCGGGGACCCTTTG,37,19,58220669,GAAAGTCGGTTTTCTGAGCTCTTACTGGCTTAGTAGCGTGGCGTTCAACG,R,ZNF154;ZNF154;ZNF154,NM_001085384;NR_110974;NR_110975,TSS200;TSS200;TSS200,chr19:58220189-58220517,S_Shore,,,,,19:62911854-62912586,,,ZNF154;ZNF154;ZNF154,ENST00000512439.2;ENST00000451275.1;ENST00000426889.1,TSS200;TSS200;TSS200,ZNF154;ZNF154;ZNF154,ENST00000317656.4;ENST00000451275.1;ENST00000426889.1,TSS200;TSS200;TSS200,chr19:58219905-58220755,3,,,,,,,19,62912481,rs181933446,41,0.000200, |
|  | 89776172 | − 0.43 | 0.0013 | cg23258188,cg23258188,0089776172,ATATATTACCRACRCAACACCAAACCACTTACAAAAATACTACTACATTC,,,II,,,GCACCTGAGCTGTGTGTTACCGGCGCAGCACCAGGCCACTTGCAGAAGTACTGCTGCATT[CG]CCAGCAAGGGCCGCCATCACGATGCGCCTCGCAGACCGCAGGGCTTGAGCAACAGGAACT,37,1,3075846,TGTGTTACCGGCGCAGCACCAGGCCACTTGCAGAAGTACTGCTGCATTCG,R,PRDM16;PRDM16,NM_022114;NM_199454,Body;Body,chr1:3071899-3072239,S_Shelf,,,,,,,,,,,,,,chr1:3075665-3076090,3,,,,,,TRUE,1,3065706,,,, |
|  | 90682536 | 0.52 | 7e−05 | cg05661282,cg05661282,0090682536,CAAAATAACCACTACCATCAAACTCTACAAATAAAACTAAACCAAAAACA,0039652823,CAAAATAACCGCTACCATCAAACTCTACGAATAAAACTAAACCGAAAACG,I,A,Red,AGTTGGCGTCCTCAGAGTGGCCGCTGCCATCAGACTCTGCGGGTAGAGCTGGGCCGGGAG[CG]ACGGGCGACATTGGTAGGGACCCGGGGACAGCGGTCCCTATCCCAGGCCTGACGTGGGTC,37,19,58220370,CGCTCCCGGCCCAGCTCTACCCGCAGAGTCTGATGGCAGCGGCCACTCTG,R,ZNF154;ZNF154,NM_001085384;NM_001085384,5'UTR;1stExon,chr19:58220189-58220517,Island,,,,,19:62911854-62912586,,,ZNF154;ZNF154;ZNF154;ZNF154;ZNF154;ZNF154,ENST00000512439.2;ENST00000451275.1;ENST00000426889.1;ENST00000512439.2;ENST00000426889.1;ENST00000451275.1,1stExon;1stExon;1stExon;5'UTR;5'UTR;3'UTR,ZNF154;ZNF154;ZNF154;ZNF154;ZNF154;ZNF154;ZNF154,ENST00000317656.4;ENST00000451275.1;ENST00000426889.1;ENST00000512439.2;ENST00000426889.1;ENST00000317656.4;ENST00000451275.1,1stExon;1stExon;1stExon;5'UTR;5'UTR;3'UTR;3'UTR,chr19:58219905-58220755,3,,,,,,TRUE,19,62912182,rs10559485;rs369658700,3;1,0.030351;0.000799, |
|  | 81781495 | 0.43 | 0.0016 | cg15746696,cg15746696,0081781495,TATTACCTAAAAAACTACATTACCCAAAAAACTCAACCTCRAATAATAAC,,,II,,,GGACCCATTGTCACGTGCACACAGGAAACGCCTTTATTCTGAGCCCTCAGAGGTCTTCTA[CG]CTATCATTCGAGGCTGAGTTTTCTGGGTAATGTAGTTCCCTAGGCAACAAGGAGGGTAAA,37,19,58400494,CGCTATCATTCGAGGCTGAGTTTTCTGGGTAATGTAGTTCCCTAGGCAAC,F,ZNF814,NM_001144989,TSS200,chr19:58399945-58400276,S_Shore,,,,,19:63091758-63092317,19:58399513-58400623,NonGene_Associated,ZNF814,ENST00000435989.1,TSS200,ZNF814,ENST00000435989.1,TSS200,chr19:58399545-58400615,3,,,,,,TRUE,19,63092306,rs530143271;rs540912247,25;51,0.000200;0.000799, |
|  | 6809506 | 0.54 | 3.2e−05 | cg15759937,cg15759937,0006809506,AAAAACTCCTAAACRCTAAATCCRCACTATAATAACTAAACCCAAAAATC,,,II,,,CGCTGGGCGCCGTCACAGAGCTCCAGAGTAGCCTCTGTGCAGCGGAGGACAACTGCTCCC[CG]ACTTCTGGGTTCAGTCACCACAGTGCGGACCTAGCGCTCAGGAGCCTCTCCTACAAATAA,37,19,58400325,GAGGCTCCTGAGCGCTAGGTCCGCACTGTGGTGACTGAACCCAGAAGTCG,F,ZNF814;ZNF814,NM_001144989;NM_001144989,1stExon;5'UTR,chr19:58399945-58400276,S_Shore,,,,,19:63091758-63092317,19:58399513-58400623,NonGene_Associated,ZNF814;ZNF814,ENST00000435989.1;ENST00000435989.1,1stExon;5'UTR,ZNF814;ZNF814,ENST00000435989.1;ENST00000435989.1,1stExon;5'UTR,chr19:58399545-58400615,3,,,,,,TRUE,19,63092137,rs540447770,43,0.000399, |
|  | 40784534 | 0.47 | 0.00038 | cg22510337,cg22510337,0040784534,TACAAAAAAAACCCCATTTCTAACACCCATAAAATACAATAAAAAAACCA,0074733139,TACGAAAAAAACCCCATTTCTAACACCCGTAAAATACGATAAAAAAACCG,I,A,Red,TTCCATCCTTCTGGCTCAGTTCACTTCCAGGCCTTTGCCCGCGCCAGTCCCTGTACCTGC[CG]GTCTCCCCACCGCATCCCACGGGTGTCAGAAATGGGGCCCCTCCCGCAAGCGCCTCAGTG,37,19,58399967,TGCGGGAGGGGCCCCATTTCTGACACCCGTGGGATGCGGTGGGGAGACCG,F,ZNF814,NM_001144989,Body,chr19:58399945-58400276,Island,,,,,19:63091758-63092317,19:58399513-58400623,NonGene_Associated,,,,,,,chr19:58399545-58400615,3,,,,,,TRUE,19,63091779,rs575261071;rs537821271;rs554271512,8;22;24,0.000200;0.000200;0.000399, |
|  | 47763189 | − 0.42 | 0.002 | cg12149490,cg12149490,0047763189,ACAATTCATAAAAATAATCTACTTAAAACCATTATACTACCTTACCATCC,,,II,,,TCCAACCAGAAGCAATTCATAAAAATGATCTGCTTAAAACCATTGTGCTGCCTTGCCATC[CG]TTGTCAAAGGTGTGAGGGGTGTGTGTGCACATGTGTTCAGTGTGGGGTGTGCGCACATGT,37,1,3062980,CGGATGGCAAGGCAGCACAATGGTTTTAAGCAGATCATTTTTATGAATTG,R,PRDM16;PRDM16,NM_199454;NM_022114,Body;Body,chr1:3059050-3059268,S_Shelf,,,,,,,,,,,,,,,,chr1:3060367-3066903,6,chr1:3062918-3063852,4,,,1,3052840,rs567083445,1,0.000200, |
|  | 43725908 | − 0.42 | 0.0018 | cg05075921,cg05075921,0043725908,ATAAACAAAAATAACCTCACTATCTACATAACTAATAACCCAACCAAAAC,,,II,,,ACGTGCTTCACTCACCCGCCCCACCCGCTCCCGGTCTCTCACTTCCACCCCTGTAGAAAG[CG]CTCTGGCTGGGCTATCAGCTATGCAGACAGTGAGGTCATTCCTGCCCACCGAGGCTCCCG,37,1,3062287,CGCTCTGGCTGGGCTATCAGCTATGCAGACAGTGAGGTCATTCCTGCCCA,F,PRDM16;PRDM16,NM_199454;NM_022114,Body;Body,chr1:3059050-3059268,S_Shelf,,,,,,,,,,,,,,,,chr1:3060367-3066903,6,,,,,1,3052147,rs564981467;rs575276218;rs117130095;rs138160212;rs112309464;rs189438930,0;23;28;40;46;51,0.000200;0.000200;0.024561;0.000399;0.017772;0.000998, |
|  | 73668587 | 0.46 | 0.00053 | cg27049766,cg27049766,0073668587,ACCATTTTAACTTCTCTAAAATATATTCACCRAATCAAAAATAACAAAAC,,,II,,,TAGACGCTTTCGTGCAGGAGGGACGACGACTCCCCTCACGCCTTCGTGGCCCCAACTCGG[CG]CTCTGCTATCTCTGATCCGGTGAACACACCTCAGAGAAGCTAAAATGGCCGCCACGAAGA,37,19,58220516,CCATTTTAGCTTCTCTGAGGTGTGTTCACCGGATCAGAGATAGCAGAGCG,F,ZNF154;ZNF154,NM_001085384;NM_001085384,5'UTR;1stExon,chr19:58220189-58220517,Island,,,,,19:62911854-62912586,,,ZNF154;ZNF154;ZNF154;ZNF154;ZNF154;ZNF154,ENST00000512439.2;ENST00000451275.1;ENST00000426889.1;ENST00000512439.2;ENST00000426889.1;ENST00000451275.1,1stExon;1stExon;1stExon;5'UTR;5'UTR;3'UTR,ZNF154;ZNF154;ZNF154;ZNF154;ZNF154;ZNF154;ZNF154,ENST00000317656.4;ENST00000451275.1;ENST00000426889.1;ENST00000512439.2;ENST00000426889.1;ENST00000317656.4;ENST00000451275.1,1stExon;1stExon;1stExon;5'UTR;5'UTR;3'UTR;3'UTR,chr19:58219905-58220755,3,,,,,,TRUE,19,62912328,rs572379115;rs187039120,9;50,0.000200;0.002596, |
|  | 99694881 | 0.52 | 7.2e−05 | cg03142586,cg03142586,0099694881,AAAAACCAACACATACAAAAATTAAAACACAACTAAACCAAAAAAATTCA,0099749976,AAAAACCGACGCGTACAAAAATTAAAACGCGACTAAACCAAAAAAATTCG,I,A,Red,AGTACAACCCTAGGAGTAACTCCGCCTCATATTCTTCGTTCCCTGCAGAAAACAGCTTTC[CG]AATTCTCCTGGCTCAGTCGCGCCTCAACCTTTGCACGCGCCGGTTCCTCCGCCTGTCACG,37,19,58220080,AGGAACCGGCGCGTGCAAAGGTTGAGGCGCGACTGAGCCAGGAGAATTCG,F,ZNF154,NM_001085384,Body,chr19:58220189-58220517,N_Shore,,,,,19:62911854-62912586,,,ZNF154,ENST00000451275.1,3'UTR,ZNF154;ZNF154,ENST00000317656.4;ENST00000451275.1,3'UTR;3'UTR,chr19:58219905-58220755,3,,,,,,TRUE,19,62911892,rs552434996;rs115903356;rs375066767,21;28;48,0.001198;0.005391;0.001997, |
|  | 7625948 | 0.51 | 0.00011 | cg02280912,cg02280912,0007625948,TCTCCRCTCAAATAATTATAAAACCTTCTATATCCTCAAATCACCTCATC,,,II,,,CGCCGGCGTCCGGGCTGCAGAGCCGTGAACAGGCGCTGCTACCTCGCTGCTTTTGGGTGA[CG]ATGAGGTGACCTGAGGACACAGAAGGCCCCACAATTACCTGAGCGGAGAGCCTCAGCGTA,37,19,58400095,CTCCGCTCAGGTAATTGTGGGGCCTTCTGTGTCCTCAGGTCACCTCATCG,F,ZNF814,NM_001144989,Body,chr19:58399945-58400276,Island,,,,,19:63091758-63092317,19:58399513-58400623,NonGene_Associated,,,,,,,chr19:58399545-58400615,3,,,,,,TRUE,19,63091907,rs75177271;rs375550431,4;23,0.001997;0.000599, |
|  | 50709158 | 0.44 | 0.0011 | cg27112264,cg27112264,0050709158,CAAAAAACRCCTTTATTCTAAACCCTCAAAAATCTTCTACRCTATCATTC,,,II,,,TCACGTGCACACAGGAAACGCCTTTATTCTGAGCCCTCAGAGGTCTTCTACGCTATCATT[CG]AGGCTGAGTTTTCTGGGTAATGTAGTTCCCTAGGCAACAAGGAGGGTAAAGGGCGCAATT,37,19,58400504,AGGAAACGCCTTTATTCTGAGCCCTCAGAGGTCTTCTACGCTATCATTCG,R,ZNF814,NM_001144989,TSS200,chr19:58399945-58400276,S_Shore,,,,,19:63091758-63092317,19:58399513-58400623,NonGene_Associated,ZNF814,ENST00000435989.1,TSS200,ZNF814,ENST00000435989.1,TSS200,chr19:58399545-58400615,3,,,,,,TRUE,19,63092316,rs544418588;rs193293655,43;17,0.000200;0.000200, |
|  | 47611970 | 0.43 | 0.0014 | cg03234186,cg03234186,0047611970,CRAAATCTATAAAAACTACATTACCTAAAATACTCTACRTTAAACRCCAC,,,II,,,TCCTTTCTTTTTGTGACTCTCAAGGAAAGTCGGTTTTCTGAGCTCTTACTGGCTTAGTAG[CG]TGGCGTTCAACGCAGAGCATTCTAGGTAATGTAGTTTTCATAGATCCCGAGGTGGGTGCC,37,19,58220657,GGGATCTATGAAAACTACATTACCTAGAATGCTCTGCGTTGAACGCCACG,F,ZNF154,NM_001085384,TSS200,chr19:58220189-58220517,S_Shore,,,,,19:62911854-62912586,,,ZNF154;ZNF154;ZNF154,ENST00000512439.2;ENST00000451275.1;ENST00000426889.1,TSS200;TSS200;TSS200,ZNF154;ZNF154;ZNF154,ENST00000317656.4;ENST00000451275.1;ENST00000426889.1,TSS200;TSS200;TSS200,chr19:58219905-58220755,3,,,,,,TRUE,19,62912469,rs150322520,46,0.001597, |
|  | 20736918 | − 0.41 | 0.0028 | cg10965478,cg10965478,0020736918,CAATCTTACTAAACTCCAACCCCAAATTAATTTAATAAAAACTCRAACTC,,,II,,,TACCTGAAAACCAGTCTTACTGAGCTCCAACCCCAAATTAATTTGGTGAGAACTCGAACT[CG]GCTATTAACCTTAATTCTGAGAGAAGAATGACACCCCCCTCCCACTGCGAGGGTCCACTG,37,15,31617633,AGTCTTACTGAGCTCCAACCCCAAATTAATTTGGTGAGAACTCGAACTCG,R,KLF13,NM_015995,TSS1500,chr15:31617763-31620908,N_Shore,,,,,,,,KLF13,ENST00000307145.3,TSS1500,KLF13,ENST00000307145.3,TSS1500,chr15:31617600-31618555,3,,,,,,TRUE,15,29404925,rs556452198,2,0.000200, |
|  | 89629166 | − 0.41 | 0.0026 | cg13084877,cg13084877,0089629166,AAAAACCAATCTTACTAAACTCCAACCCCAAATTAATTTAATAAAAACTC,,,II,,,GTCGGTTACCTGAAAACCAGTCTTACTGAGCTCCAACCCCAAATTAATTTGGTGAGAACT[CG]AACTCGGCTATTAACCTTAATTCTGAGAGAAGAATGACACCCCCCTCCCACTGCGAGGGT,37,15,31617627,CGAGTTCTCACCAAATTAATTTGGGGTTGGAGCTCAGTAAGACTGGTTTT,R,KLF13;KLF13,NM_001302461;NM_015995,TSS1500;TSS1500,chr15:31617763-31620908,N_Shore,,,,,,,,KLF13,ENST00000307145.3,TSS1500,KLF13,ENST00000307145.3,TSS1500,chr15:31617600-31618555,3,,,,,,,15,29404919,,,, |
|  | 15664924 | −0.4 | 0.0033 | cg24612696,cg24612696,0015664924,AAAAACTTTTTACCCRAAACAAAACTTTATAAAAATAACAATAACATCRC,,,II,,,CTCTGAAACAAAGAGGCTTTTTACCCGGAACAAGGCTTTATAAAAATGACAGTGGCATCG[CG]AGTCATTCAGCGCCTGCCCGGGGCGATGGAGGCGGCGAGGAAATACAGGATGCGGGCTCT,37,1,3089891,CGCGATGCCACTGTCATTTTTATAAAGCCTTGTTCCGGGTAAAAAGCCTC,R,PRDM16;PRDM16,NM_022114;NM_199454,Body;Body,,,,,,,1:3079749-3080214,,,,,,,,,,,chr1:3084897-3092440,6,,,,TRUE,1,3079751,rs17390062;rs532630229;rs552300491,44;36;6,0.031949;0.000200;0.000399, |
|  | 2764483 | −0.54 | 3.3e−05 | cg16030758,cg16030758,0002764483,CACRAACACCTAAAATACAAAACTAAAACTAACTATACAAATACTTATTC,,,II,,,CCGGCTGGGAGCACGGGCACCTGGGGTGCAGAGCTGGGGCTGGCTGTGCAGGTGCTTGTT[CG]TGTCAAGAAGCAGTTGGACTTCTCTGTGAGATTCTGCAGATCTCCCCTGTGACTGTCAAG,37,15,31653946,ACGGGCACCTGGGGTGCAGAGCTGGGGCTGGCTGTGCAGGTGCTTGTTCG,R,KLF13;KLF13,NM_015995;NM_001302461,Body;Body,,,,chr15:31653483-31653483,,,,15:31653131-31654123,Promoter_Associated,,,,KLF13,ENST00000558921.1,5'UTR,chr15:31653025-31654295,3,,,,,,,15,29441238,rs550518078;rs570613126,49;2,0.001198;0.000200, |
| XPNPEP1 | 53694927 | 0.41 | 0.0027 | cg08812189,cg08812189,0053694927,TAAACRCTTTAAACAAATAACTACCTCCCAACRCTCTAAATTAAAAAACC,,,II,,,AAGAAGGTTTCTGGGCGCTTTAAACAAATGGCTGCCTCCCAGCGCTCTGAGTTAAGGGAC[CG]GCTACCTAGCGTCTAGCTGAGGAGGAAGACGCGCAGCTGGAGAACTGTTGCCTTTGTAGT,37,3,147110367,GGGCGCTTTAAACAAATGGCTGCCTCCCAGCGCTCTGAGTTAAGGGACCG,R,ZIC4;ZIC4;ZIC4;ZIC4;ZIC4,NM_001168378;NR_033118;NR_033119;NM_032153;NM_001168379,Body;TSS200;TSS200;Body;Body,chr3:147108511-147111703,Island,,,,TRUE,3:148591852-148593286,,,ZIC4,ENST00000472749.2,TSS200,ZIC4;ZIC4;ZIC4;ZIC1;ZIC4;ZIC1;ZIC4;ZIC4;ZIC4,ENST00000494569.1;ENST00000464502.1;ENST00000494569.1;ENST00000472523.1;ENST00000472749.2;ENST00000488404.1;ENST00000475502.1;ENST00000463850.1;ENST00000493664.1,3'UTR;3'UTR;1stExon;TSS1500;TSS200;TSS1500;TSS200;TSS200;3'UTR,chr3:147109660-147110775,3,,,,,,TRUE,3,148593057,rs545170605,44,0.000200, |
|  | 42722571 | 0.45 | 0.00084 | cg15105326,cg15105326,0042722571,ATATACTTACACAACAAACTAAAATACATATAACACTTATCACAACCCCA,0091777103,ATATACTTACGCAACGAACTAAAATACGTATAACACTTATCGCAACCCCG,I,C,Grn,CCGTGCACCTTCATGTGCTTACGCAGCGAGCTGGGGTGCGTGTAGCACTTGTCGCAGCCC[CG]CACCTTGCACGTGTATGGCTTGTCGCTAGTGTGCACGTGCGAATGCTTCTTACGGTCGCT,37,3,147108916,CGGGGCTGCGACAAGTGCTACACGCACCCCAGCTCGCTGCGTAAGCACAT,R,ZIC4;ZIC4;ZIC4;ZIC4;ZIC4,NM_001168378;NR_033119;NM_032153;NR_033118;NM_001168379,Body;Body;Body;Body;Body,chr3:147108511-147111703,Island,,,,,3:148591202-148591799,,,ZIC4,ENST00000472749.2,3'UTR,ZIC4;ZIC4;ZIC4;ZIC4;ZIC4;ZIC4;ZIC4,ENST00000472749.2;ENST00000494569.1;ENST00000463850.1;ENST00000464502.1;ENST00000475502.1;ENST00000494569.1;ENST00000493664.1,3'UTR;3'UTR;3'UTR;3'UTR;3'UTR;1stExon;3'UTR,chr3:147108725-147109115,3,,,,,,TRUE,3,148591606,rs75921971,36,0.005591, |
|  | 3664217 | 0.54 | 3.8e−05 | cg23189410,cg23189410,0003664217,CTCAATTAACCTATCTTTACCTAACACAAAATCTATTCAACAACTACACA,0064654282,CTCGATTAACCTATCTTTACCTAACGCAAAATCTATTCAACAACTACGCG,I,T,Red,TTCAACAATCATTTTAATATATAGTCAATGGCTCTTTGTGGAAGGGACAAAAAGAAACTA[CG]CGCAGTTGTTGAATAGACTTTGCGCTAGGCAAAGACAGGTTAATCGAGGGCCGCATCGCG,37,3,147125712,CGCGCAGTTGTTGAATAGACTTTGCGCTAGGCAAAGACAGGTTAATCGAG,F,ZIC4;ZIC1,NM_032153;NM_003412,TSS1500;TSS1500,chr3:147126988-147128999,N_Shore,,,RDMR,TRUE,,,,ZIC4;ZIC4;ZIC1,ENST00000491672.1;ENST00000383075.3;ENST00000282928.4,TSS1500;TSS1500;TSS1500,ZIC4;ZIC4;ZIC4;ZIC4;ZIC1;ZIC1,ENST00000463250.1;ENST00000491672.1;ENST00000464144.1;ENST00000383075.3;ENST00000282928.4;ENST00000472523.1,TSS1500;TSS1500;TSS1500;TSS1500;TSS1500;5'UTR,chr3:147124905-147126215,3,,,,,,TRUE,3,148608402,,,, |
|  | 35779210 | 0.52 | 8.4e−05 | cg17546247,cg17546247,0035779210,ACCCTCRATTAACCTATCTTTACCTAACRCAAAATCTATTCAACAACTAC,,,II,,,CAACAATCATTTTAATATATAGTCAATGGCTCTTTGTGGAAGGGACAAAAAGAAACTACG[CG]CAGTTGTTGAATAGACTTTGCGCTAGGCAAAGACAGGTTAATCGAGGGCCGCATCGCGAA,37,3,147125714,CCCTCGATTAACCTGTCTTTGCCTAGCGCAAAGTCTATTCAACAACTGCG,F,ZIC4;ZIC1,NM_032153;NM_003412,TSS1500;TSS1500,chr3:147126988-147128999,N_Shore,,,RDMR,TRUE,,,,ZIC4;ZIC4;ZIC1,ENST00000491672.1;ENST00000383075.3;ENST00000282928.4,TSS1500;TSS1500;TSS1500,ZIC4;ZIC4;ZIC4;ZIC4;ZIC1;ZIC1,ENST00000463250.1;ENST00000491672.1;ENST00000464144.1;ENST00000383075.3;ENST00000282928.4;ENST00000472523.1,TSS1500;TSS1500;TSS1500;TSS1500;TSS1500;5'UTR,chr3:147124905-147126215,3,,,,,,TRUE,3,148608404,,,, |
|  | 85616592 | 0.41 | 0.0028 | cg03900143,cg03900143,0085616592,TAAAAACAACTATCCACACCAAAAAAATTCACTTTATAACAATCACAACA,0045629949,TAAAAACGACTATCCGCACCGAAAAAATTCACTTTATAACGATCACAACG,I,A,Red,CCAGGCCGAGCGCGGTTGCTGGCCCGCGCCTCCCTCCCCGAGGCACCATTGTTCCGGGAT[CG]CTGTGACCGCCACAAAGTGAATCCTTTCGGTGCGGACAGTCGCCTTCAAAGCCAGGCCCC,37,3,147111660,CGCTGTGACCGCCACAAAGTGAATCCTTTCGGTGCGGACAGTCGCCTTCA,F,ZIC4;ZIC4;ZIC4;ZIC4;ZIC4,NM_001168378;NR_033119;NM_032153;NM_001168379;NR_033118,Body;TSS1500;Body;Body;TSS1500,chr3:147108511-147111703,Island,,,,,3:148593633-148594412,,,ZIC4,ENST00000472749.2,TSS1500,ZIC4;ZIC4;ZIC4;ZIC4;ZIC1;ZIC1;ZIC4;ZIC4;ZIC4;ZIC1,ENST00000464502.1;ENST00000472749.2;ENST00000475502.1;ENST00000463850.1;ENST00000472523.1;ENST00000488404.1;ENST00000464502.1;ENST00000493664.1;ENST00000494569.1;ENST00000472523.1,3'UTR;TSS1500;TSS1500;TSS1500;1stExon;TSS200;1stExon;3'UTR;TSS1500;5'UTR,chr3:147111360-147111875,3,,,,,,TRUE,3,148594350,rs538735167;rs16859414,22;34,0.000200;0.047324, |
|  | 58709844 | 0.46 | 0.00052 | cg16790847,cg16790847,0058709844,TCCTAAAAACTACRACCTACTCCCCAAAAAACTAATTACTACTCTTATTC,,,II,,,AGGGGCCAGAATCCTAGGAGCTGCGGCCTGCTCCCCAGAGAGCTAGTTGCTGCTCTTGTT[CG]CATTGGAGATAAAGAAAGCCAAGTCCCGAACCCACTGCTGGCCGCGCGCAGAGAGCAGAT,37,3,147123429,CCTAGGAGCTGCGGCCTGCTCCCCAGAGAGCTAGTTGCTGCTCTTGTTCG,R,ZIC4;ZIC4;ZIC4,NM_032153;NM_001168378;NM_001168379,5'UTR;TSS1500;TSS200,chr3:147126988-147128999,N_Shelf,,,,,,,,ZIC4;ZIC4;ZIC4;ZIC4,ENST00000491672.1;ENST00000383075.3;ENST00000525172.2;ENST00000425731.3,5'UTR;5'UTR;TSS1500;TSS200,ZIC4;ZIC4;ZIC4;ZIC4;ZIC4;ZIC4;ZIC4;ZIC1,ENST00000462748.2;ENST00000463250.1;ENST00000491672.1;ENST00000383075.3;ENST00000525172.2;ENST00000464144.1;ENST00000425731.3;ENST00000472523.1,5'UTR;5'UTR;5'UTR;5'UTR;TSS1500;3'UTR;TSS200;5'UTR,chr3:147123325-147123735,3,,,,,,TRUE,3,148606119,,,, |
|  | 98731966 | 0.42 | 0.0017 | cg12892506,cg12892506,0098731966,CATACATAATACTAAAAATTTTAAAACAAAAACTCAAAATCACAATACCA,0049639227,CGTACATAATACTAAAAATTTTAAAACAAAAACTCGAAATCGCGATACCG,I,T,Red,GCGTGACCAGCTCGTGCATGGTGCTGAAAGTTTTGGAGCAGAGGCTCGGGGTCGCGGTGC[CG]TCGGCCGCCAGCCACTTGCAGATGAGCTCCTGTTTGATGGGCTGGCGCATGTAGCGGAAG,37,3,147113918,CGGCACCGCGACCCCGAGCCTCTGCTCCAAAACTTTCAGCACCATGCACG,R,ZIC4;ZIC4;ZIC4,NM_001168378;NM_032153;NM_001168379,Body;Body;Body,chr3:147113608-147114479,Island,,,RDMR,TRUE,3:148596274-148597489,,,,,,ZIC4;ZIC4;ZIC1,ENST00000493664.1;ENST00000493664.1;ENST00000472523.1,1stExon;3'UTR;5'UTR,chr3:147113700-147114175,3,,,,,,TRUE,3,148596608,rs535827867;rs200350429,18;15,0.000200;0.001000, |
|  | 77611150 | 0.43 | 0.0013 | cg17003736,cg17003736,0077611150,CCTTTCAAAACTACTAAACCTCTAAAATAAAAATCTAAATCTTTCACAAC,,,II,,,AAGAGGCGGCGTTGGGCTAGGCCCCTGCAGCCCGCTCGGAGCGTCCTAGGCCCGGGGCTG[CG]CTGTGAAAGACCCAGATTCTCATCCCAGAGGCCCAGCAGTCCTGAAAGGCCTCCTCTCCG,37,3,147111308,CTTTCAGGACTGCTGGGCCTCTGGGATGAGAATCTGGGTCTTTCACAGCG,F,ZIC4;ZIC4;ZIC4;ZIC4;ZIC4,NM_001168378;NR_033119;NM_032153;NM_001168379;NR_033118,Body;TSS1500;Body;Body;TSS1500,chr3:147108511-147111703,Island,,,,,3:148593633-148594412,,,ZIC4,ENST00000472749.2,TSS1500,ZIC4;ZIC1;ZIC4;ZIC4;ZIC4;ZIC1;ZIC4;ZIC4;ZIC1,ENST00000464502.1;ENST00000488404.1;ENST00000472749.2;ENST00000475502.1;ENST00000463850.1;ENST00000472523.1;ENST00000493664.1;ENST00000494569.1;ENST00000472523.1,3'UTR;TSS1500;TSS1500;TSS1500;TSS1500;1stExon;3'UTR;TSS1500;5'UTR,chr3:147110960-147111355,3,,,,,,TRUE,3,148593998,rs544535779;rs560827243,9;50,0.000200;0.000200, |
|  | 8602292 | 0.51 | 0.00013 | cg18930354,cg18930354,0008602292,AAATCTCTTCTAAAATAAATCATTACTCAAAATACCTCTCCCTACAACAC,,,II,,,AGCGATGAAATAATTTAAGGATGCGCAGCCGATGCACATTGTGTGTGCATAAAGTGGATT[CG]TGCTGCAGGGAGAGGTATTCTGAGCAATGATTCACTTCAGAAGAGATTTTTACAGGAATG,37,3,147109629,CGTGCTGCAGGGAGAGGTATTCTGAGCAATGATTCACTTCAGAAGAGATT,F,ZIC4;ZIC4;ZIC4;ZIC4;ZIC4,NM_001168378;NR_033119;NM_032153;NR_033118;NM_001168379,Body;Body;Body;Body;Body,chr3:147108511-147111703,Island,,,,,3:148591852-148593286,,,ZIC4,ENST00000472749.2,3'UTR,ZIC4;ZIC4;ZIC4;ZIC4;ZIC4;ZIC4;ZIC4,ENST00000472749.2;ENST00000494569.1;ENST00000463850.1;ENST00000464502.1;ENST00000475502.1;ENST00000494569.1;ENST00000493664.1,3'UTR;3'UTR;3'UTR;3'UTR;3'UTR;1stExon;3'UTR,,,chr3:147108278-147112699,6,chr3:147104865-147116795,6,,TRUE,3,148592319,rs545334995,1,0.000200, |
|  | 36628967 | 0.43 | 0.0014 | cg22203776,cg22203776,0036628967,AAAAAAAACCCTATCATTAAAAATAAATTCCTTCTCCAACTCAAAACTAC,,,II,,,AAAAAAAAAAAAAAAAAAACCCTGTCATTAAAGATGAGTTCCTTCTCCAGCTCAGGACTG[CG]AGTCTACCTTCCGGGTGTCCGCCGCCAGCTCCCGGAGTAGCAGGCCTGGGGCCAGGATTC,37,3,147112316,CGCAGTCCTGAGCTGGAGAAGGAACTCATCTTTAATGACAGGGTTTTTTT,R,ZIC4;ZIC4;ZIC4,NM_001168378;NM_032153;NM_001168379,Body;Body;Body,chr3:147113608-147114479,N_Shore,,,RDMR,,3:148595006-148595248,,,,,,ZIC4;ZIC4;ZIC1,ENST00000493664.1;ENST00000464502.1;ENST00000472523.1,3'UTR;TSS1500;5'UTR,chr3:147112060-147112655,3,,,,,,TRUE,3,148595006,rs9811502,43,0.037500, |
|  | 9791841 | 0.46 | 6e−04 | cg20939084,cg20939084,0009791841,TCTCCAAAAAACCTCTAAAACAACRTAATAAAATATTATTACTAACRAAC,,,II,,,GGCAAACATTTAGCAGCATTCTTCAAATCTTGCCTAAACCTTCCGGGATCCCTCCAGATA[CG]CTCGCCAGTAATAATATTTCATTACGCTGCTCCAGAGGCTTCCTGGAGACCGTGCTGTGG,37,3,147109784,CGCTCGCCAGTAATAATATTTCATTACGCTGCTCCAGAGGCTTCCTGGAG,F,ZIC4;ZIC4;ZIC4;ZIC4;ZIC4,NM_001168378;NR_033119;NM_032153;NR_033118;NM_001168379,Body;Body;Body;Body;Body,chr3:147108511-147111703,Island,,,,,3:148591852-148593286,,,ZIC4;ZIC4,ENST00000472749.2;ENST00000472749.2,3'UTR;1stExon,ZIC4;ZIC4;ZIC4;ZIC4;ZIC4;ZIC4;ZIC1;ZIC4;ZIC4,ENST00000472749.2;ENST00000494569.1;ENST00000463850.1;ENST00000464502.1;ENST00000475502.1;ENST00000494569.1;ENST00000472523.1;ENST00000472749.2;ENST00000493664.1,3'UTR;3'UTR;3'UTR;3'UTR;3'UTR;1stExon;TSS1500;1stExon;3'UTR,chr3:147109660-147110775,3,,,,,,TRUE,3,148592474,rs529678587;rs58262807,35;45,0.001597;0.392772, |
|  | 86661913 | 0.44 | 0.00099 | cg00235367,cg00235367,0086661913,TAACCRATCCCTTAACTCAAAACRCTAAAAAACAACCATTTATTTAAAAC,,,II,,,AAGCCTAGATTCCTGCCGGAGCTGCAAGTGCTGCGGAAATGGGGGAAGAAGGTTTCTGGG[CG]CTTTAAACAAATGGCTGCCTCCCAGCGCTCTGAGTTAAGGGACCGGCTACCTAGCGTCTA,37,3,147110322,AGCCGGTCCCTTAACTCAGAGCGCTGGGAGGCAGCCATTTGTTTAAAGCG,F,ZIC4;ZIC4;ZIC4;ZIC4;ZIC4,NM_001168378;NR_033118;NR_033119;NM_032153;NM_001168379,Body;TSS200;TSS200;Body;Body,chr3:147108511-147111703,Island,,,,TRUE,3:148591852-148593286,,,ZIC4,ENST00000472749.2,TSS200,ZIC4;ZIC4;ZIC4;ZIC1;ZIC4;ZIC1;ZIC4;ZIC4;ZIC4,ENST00000494569.1;ENST00000464502.1;ENST00000494569.1;ENST00000472523.1;ENST00000472749.2;ENST00000488404.1;ENST00000475502.1;ENST00000463850.1;ENST00000493664.1,3'UTR;3'UTR;1stExon;TSS1500;TSS200;TSS1500;TSS200;TSS200;3'UTR,chr3:147109660-147110775,3,,,,,,TRUE,3,148593012,rs545170605,3,0.000200, |
|  | 34662265 | 0.44 | 0.001 | cg19516404,cg19516404,0034662265,CACCCTAATCCTACCTCAAAAAACTAACTACTAAAATCTACTCTCTACRC,,,II,,,TTGCTGCTCTTGTTCGCATTGGAGATAAAGAAAGCCAAGTCCCGAACCCACTGCTGGCCG[CG]CGCAGAGAGCAGATCCCAGCAGTCAGCTTCCTGAGGCAGGACCAGGGTGAGGGAGGGCAA,37,3,147123475,ACCCTGGTCCTGCCTCAGGAAGCTGACTGCTGGGATCTGCTCTCTGCGCG,F,ZIC4;ZIC4;ZIC4,NM_032153;NM_001168378;NM_001168379,5'UTR;TSS1500;TSS200,chr3:147126988-147128999,N_Shelf,,,,,,,,ZIC4;ZIC4;ZIC4;ZIC4,ENST00000491672.1;ENST00000383075.3;ENST00000525172.2;ENST00000425731.3,5'UTR;5'UTR;TSS1500;TSS200,ZIC4;ZIC4;ZIC4;ZIC4;ZIC4;ZIC4;ZIC4;ZIC4;ZIC1,ENST00000462748.2;ENST00000463250.1;ENST00000491672.1;ENST00000383075.3;ENST00000525172.2;ENST00000464144.1;ENST00000425731.3;ENST00000463250.1;ENST00000472523.1,5'UTR;5'UTR;5'UTR;5'UTR;TSS1500;3'UTR;TSS200;ExonBnd;5'UTR,chr3:147123325-147123735,3,,,,,,TRUE,3,148606165,rs184839225;rs543662244,5;10,0.000200;0.000399, |
|  | 48616842 | 0.44 | 0.00098 | cg18082337,cg18082337,0048616842,CTTCAAAACCAAATACAAACTTATAAATCACATCCACATACACACAAACA,0019783576,CTTCAAAACCAAATACAAACTTATAAATCACATCCGCGTACACACGAACG,I,A,Red,GATTTTCTGATCTAGCAAAGACCTTCCCACACCCCGGGAAAGGACAAGGGAAGGGCTTCT[CG]CCCGTGTGCACGCGGATGTGATTTACAAGTTTGTATTTGGCTTTGAAGGGCTTTCCCTGG,37,3,147113726,CGCCCGTGTGCACGCGGATGTGATTTACAAGTTTGTATTTGGCTTTGAAG,F,ZIC4;ZIC4;ZIC4,NM_001168378;NM_032153;NM_001168379,Body;Body;Body,chr3:147113608-147114479,Island,,,RDMR,TRUE,3:148596274-148597489,,,,,,ZIC4;ZIC4;ZIC1,ENST00000493664.1;ENST00000493664.1;ENST00000472523.1,1stExon;3'UTR;5'UTR,chr3:147113700-147114175,3,,,,,,TRUE,3,148596416,rs75011808,25,0.000399, |
|  | 92688890 | 0.45 | 9e−04 | cg12388007,cg12388007,0092688890,AAAAAACAAACRCTTTAAAACTAATTAAAACAAACAAAACCCCAATTCCC,,,II,,,GCCGCATCCAGGAAAAACAGGCGCTTTGGGGCTGGTTAGAACAAACAAAGCCCCAATTCC[CG]AGCCCTGTTGAGGCTCGGACAGAGAGGTTTGCGCACAACCTGCGCTTCTGCGCAATCAGC,37,3,147110499,CGGGAATTGGGGCTTTGTTTGTTCTAACCAGCCCCAAAGCGCCTGTTTTT,R,ZIC4;ZIC4;ZIC4;ZIC4;ZIC4,NM_001168378;NR_033119;NM_032153;NM_001168379;NR_033118,Body;TSS1500;Body;Body;TSS1500,chr3:147108511-147111703,Island,,,DMR,TRUE,3:148591852-148593286,,,ZIC4,ENST00000472749.2,TSS1500,ZIC4;ZIC4;ZIC4;ZIC1;ZIC1;ZIC4;ZIC4;ZIC4;ZIC4,ENST00000494569.1;ENST00000464502.1;ENST00000494569.1;ENST00000472523.1;ENST00000488404.1;ENST00000472749.2;ENST00000475502.1;ENST00000463850.1;ENST00000493664.1,3'UTR;3'UTR;1stExon;TSS1500;TSS1500;TSS1500;TSS1500;TSS1500;3'UTR,chr3:147109660-147110775,3,,,,,,TRUE,3,148593189,rs550553553;rs560955876;rs530034534,37;12;7,0.000200;0.000399;0.000200, |
| GP1BA | 54742598 | − 0.52 | 7.7e−05 | cg10493186,cg10493186,0054742598,CAACCTAACATAACAACCAAAAAAAATACCTAACTCTCAACAAAAAACCA,0002783984,CAACCTAACGTAACGACCAAAAAAAATACCTAACTCTCGACAAAAAACCG,I,C,Grn,AAAGGGCCTCTGCAGCCTGGCGTGACGGCCAGAGGAGGTGCCTGGCTCTCGGCAGGGAGC[CG]CAGGGCTCTTTCTTCCTCTCTGGTTCGTACTTCTCCTCTCTCATGGGGGTCCCCCAAAGC,37,1,3134756,CGGCTCCCTGCCGAGAGCCAGGCACCTCCTCTGGCCGTCACGCCAGGCTG,R,PRDM16;PRDM16,NM_022114;NM_199454,Body;Body,,,,,,,1:3123982-3124644,,,,,,,,,chr1:3134685-3135075,3,,,,,,TRUE,1,3124616,rs575413405;rs370514834;rs145645797,37;12;10,0.000200;0.000599;0.000599, |
|  | 3787232 | − 0.43 | 0.0015 | cg26425711,cg26425711,0003787232,ACCAAATTCCCCCCTATAACTTCAAAAAAATAAACRCCCTAAAAAAACTC,,,II,,,GTGGCAGGGAGACCAGGTTCCCCCCTGTGGCTTCAGGAGGGTGGGCGCCCTGAGAGAACT[CG]CTTCAAGGCCCCTGTCCCTGCCTGGAAGGAGCCCAGCGTGCGAATTCCAGCTGCCCTGCA,37,1,3251680,CCAGGTTCCCCCCTGTGGCTTCAGGAGGGTGGGCGCCCTGAGAGAACTCG,R,PRDM16;PRDM16,NM_022114;NM_199454,Body;Body,,,,,,,1:3241339-3241889,,,PRDM16,ENST00000512462.1,5'UTR,PRDM16;PRDM16,ENST00000512462.1;ENST00000463591.1,5'UTR;5'UTR,chr1:3251565-3252295,3,,,,,,TRUE,1,3241540,rs560315508;rs532848837;rs552220666;rs569033023;rs553936239,39;26;13;7;1,0.000200;0.000399;0.000200;0.000799;0.001198, |
|  | 88804205 | − 0.44 | 0.00097 | cg17445936,cg17445936,0088804205,CCCTAATAAAATAAATAACAATCACACATCCTCCAAACTATCATAAAACC,,,II,,,TCAGGGCCGGCCCCTGGTGGGGTGAATGGCAGTCACACATCCTCCAGGCTGTCATGGGGC[CG]AGGCTCCGTGCTCAGCAATGCGGTTCCTCTGGGTGGTTTATGCTCCCGGTGCAAAGCCTT,37,1,3090345,CGGCCCCATGACAGCCTGGAGGATGTGTGACTGCCATTCACCCCACCAGG,R,PRDM16;PRDM16,NM_022114;NM_199454,Body;Body,,,,,,,1:3079749-3080214,,,,,,,,,chr1:3090200-3090475,3,,,,,,TRUE,1,3080205,rs114106344;rs115133463,10;2,0.046725;0.001797, |
|  | 97777585 | − 0.4 | 0.0029 | cg22510139,cg22510139,0097777585,ACCAAATATTTTCACTCRATTTTACAAATCTACTCCAAATCTAAAACATC,,,II,,,AGCTGTCAGTCAAGCGTCTTTCACAGCCAGCCAAGACCTTTTGATTTCTAGAACAAACAG[CG]ATGTCTTAGACCTGGAGCAGATTTGCAAAATCGAGTGAAAACATCTGGCTGTGCTAACAA,37,1,3058822,CCAGATGTTTTCACTCGATTTTGCAAATCTGCTCCAGGTCTAAGACATCG,F,PRDM16;PRDM16,NM_022114;NM_199454,Body;Body,chr1:3059050-3059268,N_Shore,,,,,1:3048260-3049151,,,,,,,,,,,chr1:3049249-3060070,6,,,,TRUE,1,3048682,,,, |
|  | 99772122 | − 0.46 | 6e−04 | cg12473797,cg12473797,0099772122,ACTACTAAACAAAAAACAATCCRACRTAACAAAAACAAACCTTTCTCAAC,,,II,,,TATGACTGATCCTTTTTCTTCACGTAAGCGGTGGAGTTAAGTGTTTGGAATTGCTGCAGA[CG]CTGAGAAAGGCCTGCCCCTGTCACGCCGGATTGTCCCCTGCTCAGCAGCTGACACAGCCC,37,1,3036358,CGCTGAGAAAGGCCTGCCCCTGTCACGCCGGATTGTCCCCTGCTCAGCAG,F,PRDM16;PRDM16,NM_022114;NM_199454,Body;Body,chr1:3038067-3038343,N_Shore,,,,,,1:3036332-3036547,Unclassified_Cell_type_specific,,,,,,,chr1:3036340-3036655,3,,,,,,TRUE,1,3026218,rs141049593;rs545903303;rs113911649,0;29;39,0.001597;0.000200;0.006789, |
|  | 12656295 | − 0.43 | 0.0015 | cg01431482,cg01431482,0012656295,AATAAAAAAACTACAACCCCCACTAAATAAAAACTATATATATCTTACTC,,,II,,,CTAGCCCCTGGGGTGGGGGAGCTGCAGCCCCCACTAGATGGGGACTGTGTGTGTCTTGCT[CG]TGTTTGAGGGTGATGCGTGTGGAAGGTGTGCTGGAGACAGGGTAGAGGTAAGCGGGGCTG,37,1,2989085,CGAGCAAGACACACACAGTCCCCATCTAGTGGGGGCTGCAGCTCCCCCAC,R,PRDM16;PRDM16,NM_022114;NM_199454,Body;Body,chr1:2990030-2990718,N_Shore,,,,,,,,,,,,,,chr1:2988825-2989375,3,,,,,,TRUE,1,2978945,rs546014913,29,0.000799, |
|  | 70796586 | − 0.4 | 0.0031 | cg11946666,cg11946666,0070796586,AAATAAACCTAAATACRAAAAATAACAACAATTCCATCTAATAAAACCCC,,,II,,,CAGCTCACAGGAAGTGGGCCTGAATGCGGAGAATAGCAGCAATTCCATCTGATAAGGCCC[CG]CTAAACCCCACATTGACAGGCCCCACCGCCCACCCGGGCTCCAGGGGCCAAGGGAGGGGC,37,1,3247595,CGGGGCCTTATCAGATGGAATTGCTGCTATTCTCCGCATTCAGGCCCACT,R,PRDM16;PRDM16,NM_022114;NM_199454,Body;Body,,,,,,,,1:3247537-3247655,Unclassified_Cell_type_specific,PRDM16,ENST00000512462.1,5'UTR,PRDM16;PRDM16,ENST00000512462.1;ENST00000463591.1,5'UTR;5'UTR,chr1:3247425-3247715,3,,,,,,TRUE,1,3237455,rs535391853;rs76122673,44;25,0.000200;0.085064, |
|  | 63733589 | − 0.44 | 0.0012 | cg25618424,cg25618424,0063733589,CCAAAACCAAAACRACAACCTTCCCTCTAAAAACCRATTAAAACAACAAC,,,II,,,CAGTCCCCTCCCCAGAGCCAGGACGGCAGCCTTCCCTCTGAAGGCCGGTTAGGGCAGCAG[CG]CCAGGACGTCCGGCGGGAATCCAGCGTCTTGCCCCGCTTCGGACGAAAACACAGAGGCTG,37,1,2989307,CAGAGCCAGGACGGCAGCCTTCCCTCTGAAGGCCGGTTAGGGCAGCAGCG,R,PRDM16;PRDM16,NM_022114;NM_199454,Body;Body,chr1:2990030-2990718,N_Shore,,chr1:2989176-2989176,,,1:2979167-2979278,,,,,,,,,chr1:2988825-2989375,3,,,,,,TRUE,1,2979167,rs146758878;rs527703576;rs547776195;rs372464526;rs532428462,38;29;15;14;5,0.011582;0.000399;0.000599;0.002995;0.000200, |
